# Melatonin inhibits lipid accumulation to repress prostate cancer progression by mediating the epigenetic modification of CES1

**DOI:** 10.1002/ctm2.449

**Published:** 2021-06-06

**Authors:** Lijie Zhou, Cai Zhang, Xiong Yang, Lilong Liu, Junyi Hu, Yaxin Hou, Hong Tao, Haruhiko Sugimura, Zhaohui Chen, Liang Wang, Ke Chen

**Affiliations:** ^1^ Department of Urology, Union Hospital, Tongji Medical College Huazhong University of Science and Technology Wuhan China; ^2^ Shenzhen Huazhong University of Science and Technology Research Institute Shenzhen China; ^3^ Department of Clinical Laboratory the First Affiliated Hospital of Zhengzhou University Zhengzhou Henan China; ^4^ Department of Tumor Pathology Hamamatsu University School of Medicine Hamamatsu Shizuoka Japan

**Keywords:** CES1, enzalutamide resistance, lipid metabolism, melatonin, prostate cancer

## Abstract

**Background:**

Androgen deprivation therapy (ADT) is the main clinical treatment for patients with advanced prostate cancer (PCa). However, PCa eventually progresses to castration‐resistant prostate cancer (CRPC), largely because of androgen receptor variation and increased intratumoral androgen synthesis. Several studies have reported that one abnormal lipid accumulation is significantly related to the development of PCa. Melatonin (MLT) is a functionally pleiotropic indoleamine molecule and a key regulator of energy metabolism. The aim of our study is finding the links between CRPC and MLT and providing the basis for MLT treatment for CRPC.

**Methods:**

We used animal CRPC models with a circadian rhythm disorder, and PCa cell lines to assess the role of melatonin in PCa.

**Results:**

We demonstrated that MLT treatment inhibited tumor growth and reversed enzalutamide resistance in animal CRPC models with a circadian rhythm disorder. A systematic review and meta‐analysis demonstrated that MLT is positively associated with an increased risk of developing advanced PCa. Restoration of carboxylesterase 1 (CES1) expression by MLT treatment significantly reduced lipid droplet (LD) accumulation, thereby inducing apoptosis by increasing endoplasmic reticulum stress, reducing de novo intratumoral androgen synthesis, repressing CRPC progression and reversing the resistance to new endocrine therapy. Mechanistic investigations demonstrated that MLT regulates the epigenetic modification of CES1. Ces1‐knockout (Ces^−/−^) mice verified the important role of endogenous Ces1 in PCa.

**Conclusions:**

Our findings provide novel preclinical and clinical information about the role of melatonin in advanced PCa and characterize the importance of enzalutamide combined with MLT administration as a therapy for advanced PCa.

## Graphical abstract

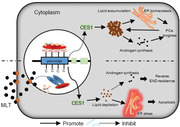

## 1 INTRODUCTION

Prostate cancer (PCa) is the second leading cause of male cancer‐related mortality.^1^ Surgery, androgen deprivation therapy (ADT) is the main clinical treatment for PCa. However, some patients eventually develop castration‐resistant prostate cancer (CRPC) due to the reactivation and abnormal activation of androgen receptor (AR).^2^ Enzalutamide, a second‐generation antiandrogen, has been approved for the treatment of CRPC; however, resistance to enzalutamide occurs frequently.^3^, ^4^

Epidemiological studies have reported that a high‐fat diet plays an important role in the development of PCa.^5^ Several studies have demonstrated that the occurrence of PCa, especially advanced PCa, is significantly related to metabolic syndrome, a clinical condition characterized by altered blood lipid levels (such as high triglycerides [TGs] and hypercholesterolemia), glucose intolerance, and obesity.^6^, ^7^ Lipid accumulation in fatty acid metabolism that is necessary for energy production, membrane synthesis, and the posttranslational modification of signaling molecules has been considered as significant factors in early stages of malignant transformation and tumor progression.^8^, ^9^ Recent studies have confirmed that the AR abnormal activation and reactivation lead to the synthesis and abnormal deposit of lipids in PCa cell.^10^ In treatment‐induced progression of PCa, certain enzymes are activated to promote the synthesis of polyunsaturated lipids, which prevent the iron‐mediated reactions of peroxides that induce ferroptotic cell death.^11^ Additionally, cholesterol, which is a precursor of androgen synthesis, is abnormally accumulated in lipid droplets and promotes an increase in endogenous androgen biosynthesis of tumor cells, which has been known as a mechanism of CRPC^12^, ^13^ and enzalutamide‐resistant PCa.^14^ In brief, metabolic alterations, especially abnormal lipid metabolism, play crucial roles in PCa development, progression, and resistance to therapy. The treatment of abnormal lipid accumulation in PCa cells may be a means to treat CRPC and enzalutamide‐resistant PCa.

**HIGHLIGHTS**

- Melatonin reduced lipid accumulation by upregulating the expression of the lipid metabolism‐related gene CES1 in PCa cell.
- CES1 was downregulated and predicted poor prognosis in PCa.
- MLT inhibited CRPC progression and reversed enzalutamide resistance by CES1.
- MLT regulated the epigenetic modification of CES1 though SIRT1‐mediated DNMT1 deacetylation.

Melatonin (MLT) is a functionally pleiotropic indoleamine molecule secreted by the pineal gland. In recent decades, epidemiological studies have demonstrated that chronic circadian rhythm disorder, leading to decrease MLT secretion, has a positive association with increased risk for many cancers.^15^, ^16^, ^17^ Tai et al reported that lower levels of melatonin sulfate in the morning are closely related to PCa,^15^ and Sigurd et al found that the lower urinary level of 6‐sulfatoxymelatonin voided first in the morning, the higher risk for advanced PCa.^16^ Several clinical trials suggest a significant positive effect of oral MLT, alone and in combination with chemotherapy, for cancer patients.^18^, ^19^, ^20^ MLT is a potent antioxidant and regulator of lipid metabolism, that reduces the levels of TGs, cholesterol, and other lipids in the blood, and has an anti‐obesity effect.^21^, ^22^, ^23^ Several studies demonstrated that MLT has an important role in the regulation of intermediary metabolism and breast cancer.^24^, ^25^, ^26^ Zharinov et al have reported that MLT has a significant positive effect on long‐term survival in PCa patients with poor prognosis. Wang et al have reported that melatonin reduced PCa metastasis by inhibiting the expression of MMP13. However, the mechanism of melatonin in CRPC, whether MLT represses PCa progression thought mediating lipid metabolism, are not quite clear, especially in enzalutamide‐resistant CPRC.

In the present study, a meta‐analysis demonstrated that low levels of MLT and high levels of TG and total cholesterol (T‐CHO) are related to the increased risk for PCa, especially advanced PCa, and higher melatonin levels correspond to lower lipid levels. We also determined that MLT inhibits lipid accumulation in PCa cells in an MT1‐dependent manner. Functionally, MLT inhibits intracellular lipid droplet accumulation and cell proliferation and migration and reduces the intratumoral androgen biosynthesis in CRPC and enzalutamide‐resistant PCa cells by promoting the expression of the lipid metabolism‐related carboxylesterase 1 (CES1) gene. Mechanistically, our investigations demonstrated that the level of CES1 expression is related to the level of CpG islands (CGIs) methylation of the CES1 promoter, and MLT decreases the methylation level of the CES1 promoter by promoting sirtuin 1 (SIRT1) expression, which mediates the deacetylation of DNA methyltransferase 1 (DNMT1).

## 2 RESULTS

### 2.1 MLT reduced lipid accumulation in PCa

**FIGURE 1:**
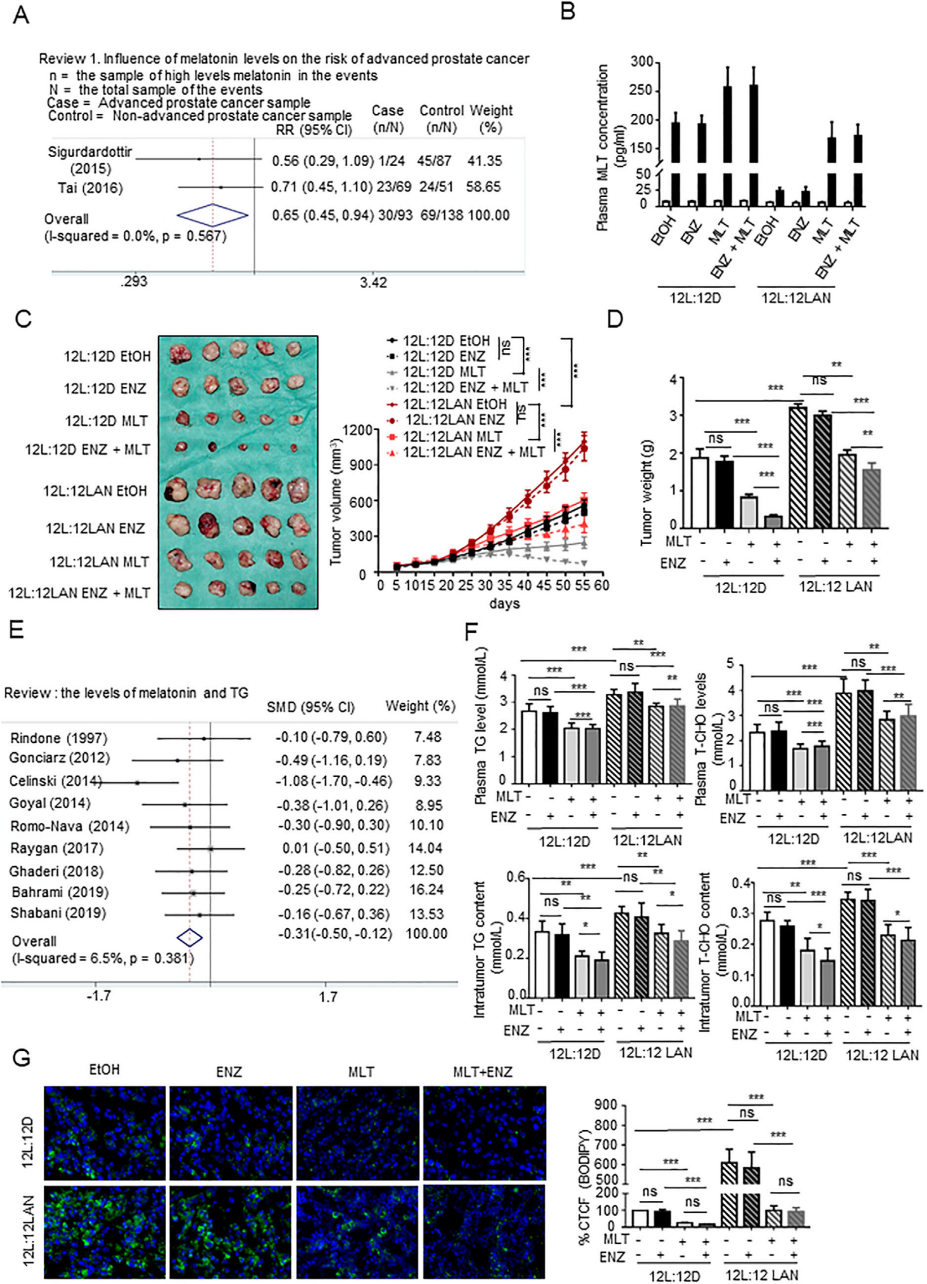
MLT reduced lipid accumulation in PCa. (A) Meta‐analysis of the melatonin levels and risk of advanced PCa. (B) The plasma MLT levels in all groups, which were assayed as described in “Materials and Methods” at 12:00 and 24:00 h. (C and D) C57BL/6 mice bearing Rm‐1 cell xenograft tumors were treated as described in “Materials and Methods”. Tumor volumes were measured every 5 days. (*n* = 5 per group). Tumors were weighed after resection. The graphs show the means ± SEM. One‐way ANOVA followed by Tukey's multiple comparison test, *α* = 0.05; *p < 0.05, **p < 0.01, and ***p < 0.001. (E) Meta‐analysis of melatonin and TG levels in the blood. (F) Assays of the TG and T‐CHO contents in the Rm‐1 tumor tissues and in the serum of all groups were used to generate indicators of intracellular lipids. The graphs present the means ± SEM. (G) The Rm‐1 tumor tissues were stained with BODIPY (green) and DAPI (blue). Representative images of BODIPY staining under each condition. Quantification of the corrected total cell fluorescence (CTCF) of BODIPY staining. The graphs present the means ± SEM. Abbreviations: ns, no significance; RR, risk ratio; SMD, standard mean difference.

To investigate whether melatonin is associated with the risk of PCa, a systematic review and meta‐analysis were performed according to a flowchart (Figure S1A). Men with lower MLT levels had increased risk of PCa (Table S1), and low melatonin levels increased the incidence of advanced PCa (Figure 1A).

To investigate the influence of circadian rhythm disorder on PCa, we used mouse‐derived prostate Rm‐1 cells to generate a PCa model with C57BL/6 mice. The levels of MLT in mice housed and exposed to LAN were significantly lower than those in the normal light rhythm group, and supplementation with MLT in drinking water increased the level of melatonin in the blood (Figure 1B), indicating that LAN‐induced circadian rhythm disorder decreased the MLT levels in these mice. Rm‐1 PCa tumors in the mice exposed to LAN showed significantly increased size, growth rate, and weight, compared to those of the tumors from the normal light rhythm group and from the LAN group receiving supplemental MLT, indicating that the LAN‐induced decrease in the level of MLT stimulated PCa growth in the mice. Comparison of the control group with the ENZ treatment group exposed to normal light or LAN indicated that the Rm‐1 xenograft tumors were not sensitive to ENZ, suggesting that Rm‐1 is a mouse‐derived CRPC cell line (Figures 1C and 1D). The result of the real‐time PCR analysis indicated positive expression of AR‐V7 in the Rm‐1 tumors (Figure S1C). The results of IHC and Tunel assays indicated that circadian rhythm disorder promoted cell proliferation and inhibited the apoptosis rate, while MLT supplementation had the opposite effect (Figure S1D). Moreover, comparison of the ENZ‐alone treatment group with ENZ + MLT treatment group indicated that MLT treatment increases the sensitivity of Rm‐1 tumors to ENZ regardless of exposure to normal light rhythm or LAN (Figures 1C and 1D).

MLT is a potent antioxidant and a key regulator of energy metabolism, especially lipid metabolism.^21^, ^22^, ^23^ Several studies have demonstrated that the occurrence and progression of PCa, especially CRPC, are significantly related to the lipid metabolism.^10^ The meta‐analysis of the association of melatonin with lipid levels demonstrated that MLT is negatively correlated with TG, T‐CHO, and LDL and positively correlated with HDL in the blood (Figures 1E, S1B, and Table S2). To investigate the influence on lipids, we assayed the TG and T‐CHO contents in the blood and tumor tissues of the groups. The results indicated that the TG and T‐CHO levels were higher in the LAN‐exposed group compared to those in the normal light rhythm group and lower than those in the MLT treatment group (Figure 1F). Similar results were shown by BODIPY staining (Figure 1G). Overall, these results indicate that MLT reduced lipid accumulation in PCa.

### 2.2 MLT upregulated the expression of the lipid metabolism‐related gene CES1 in PCa cells

**FIGURE 2:**
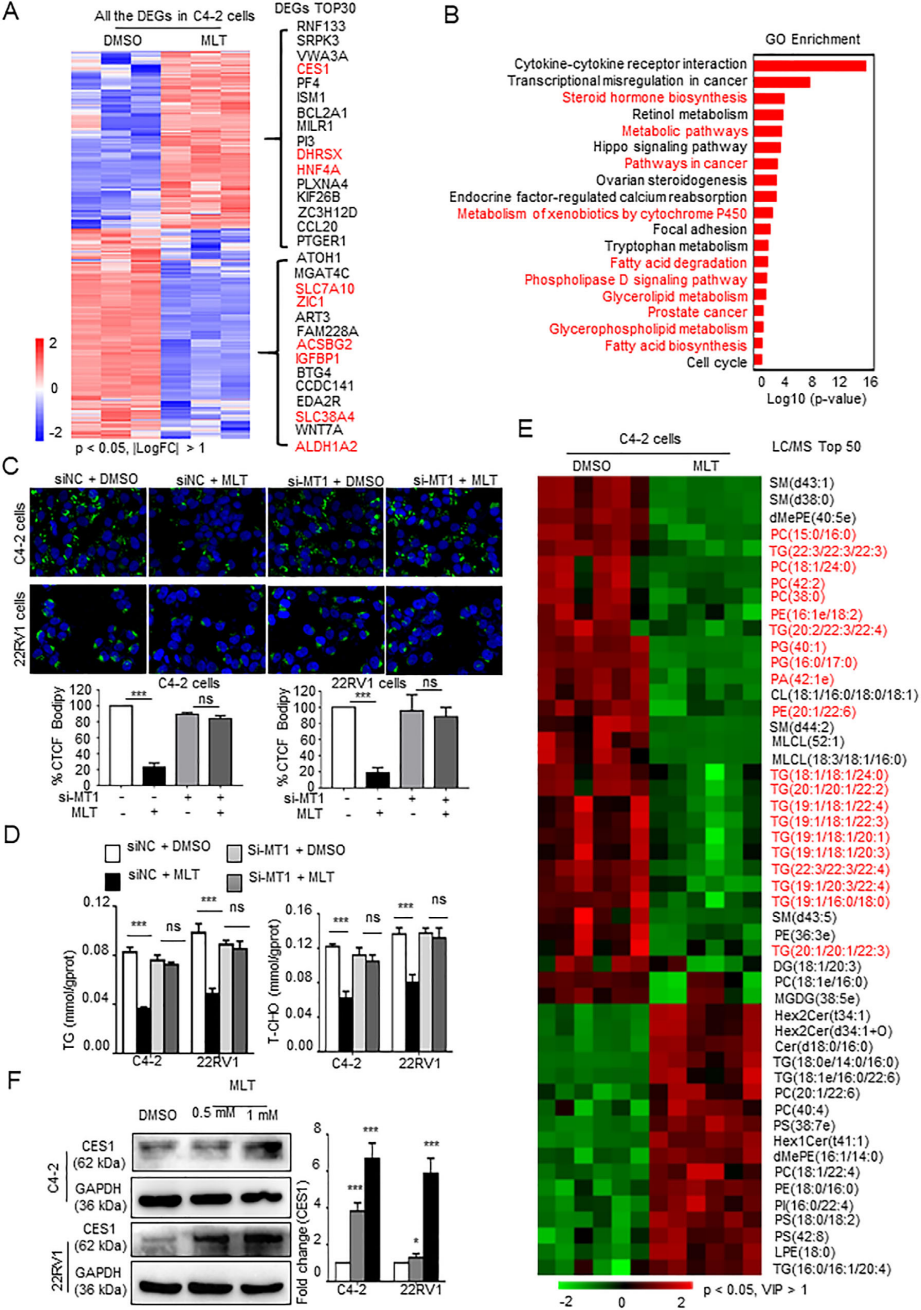
MLT promotes the expression of the lipid metabolism‐related CES1 gene. (A) Heat map of the statistically significantly differentially expressed genes (DEGs) according to the RNA sequencing data from the MLT‐treated (1 mM) C4‐2 cells versus the cells treated with vehicle control (DMSO). Heat map of top 30 DEGs identified by RNA sequencing was showed with their gene names. Red: lipid metabolism genes. *n* = 3, *p* < 0.05, |LogFC| > 1. (B) GO analysis of the DEGs indicated that MLT treatment is significantly associated with the lipid metabolism signaling pathway. (C) C4‐2 and 22RV1 cells were transfected with siMT‐1 or siNC (control). After 48 h, the cells were incubated with DMSO or MLT (1 mM) for 48 h and stained with BODIPY (green) and DAPI (blue). Representative images of BODIPY staining under each condition. Quantification of CTCF. (D) Total cell triglyceride (TG) and cholesterol (T‐CHO) contents were measured in cells treated with MLT (1 mM, 48 h) or DMSO using TG or total cholesterol assay kits. (E) Heat map of the top 50 lipid metabolites with statistically significant differences according to the LC‐MS/MS lipidomic assay performed to detect intracellular lipids in the C4‐2 cells treated with MLT (1 mM, 48 h) or DMSO (*n* = 6). Student's *t* test, *p* < 0.05, VIP > 1. (F) Detection of the CES1 expression in the C4‐2 and 22RV1 cells treated with various concentrations of MLT. Densitometry and statistical analysis. Representative images are shown

To determine the regulatory mechanisms of MLT in PCa, we performed RNA sequencing with C4‐2 cells treated with MLT (1 mM). A comparison of treated C4‐2 cells with control cells led to the identification of 1333 differently expressed genes (DEG), including 614 upregulated and 719 downregulated genes (|logFC| > 1, *p* < 0.05) in the C4‐2 cells treated with MLT (Figures 2A and S2A). Subsequently, GO analysis showed that MLT treatment mediated most of changes in lipid metabolism pathways (Figure 2B). In addition, the result of GO analysis in Figure 2B also suggested that MLT could have an influence on cytokine‐cytokine receptor interaction and transcriptional misregulation pathways in PCa.

Lipids are mainly stored in the cells as lipid droplets. A BODIPY assay was carried out to detect the lipid droplets, and the results demonstrated that treatment with MLT decreased the fluorescence in the C4‐2 and 22RV1 cells compared to that in the corresponding control cells, indicating that MLT inhibits lipid deposition in PCa cells (Figure 2C). The melatonin receptor has two subtypes, MT1 and MT2; MT1 is predominantly expressed in prostate tissue, and MT2 is negligibly expressed. To investigate whether the effect of MLT on lipid accumulation in PCa cells depends on MT1, we generated an siRNA for MT1 (Figure S2B); the knockdown of MT1 eliminated the effect of melatonin on the depletion of lipid droplets in C4‐2 and 22RV1 cells (Figure 2C). As quantitative indicators of lipid accumulation, the cholesterol and TG contents were detected, and the results demonstrated melatonin inhibits lipid accumulation, and the knockdown of MT1 eliminated the effect of melatonin on PCa cells (Figure 2D). Then, the results of the LC/MS assay demonstrated that treatment with MLT downregulated the components in lipid droplets, TG, PE, SM, and PC (Figures 2E and S2C).

To investigate the mechanism of lipid metabolism changes induced by melatonin in PCa, lipid‐related genes were identified in the top 30 DEGs based on the RNA sequencing results; three genes (DHRSX,^27^ HNF4A,^28^ and CES1^29^) were upregulated, and six genes (SLC7A10,^30^ IGFBP1,^31^ ALDH1A2,^32^ ACSBG2,^33^ ZIC1,^34^ and SLC38A4^35^) were downregulated in the C4‐2 cells treated with MLT compared with the control cells (Figure 2A). Besides, the top 30 DEGs included other functional genes, for example, CCL20^36^ and MILR1, ^37^ are related to tumor microenvironment and inflammation. The results of the Western blot assay of the levels of proteins encoded by these lipid‐related genes indicated that compared with the control cells, CES1 was the most significantly and consistently changed in the C4‐2 and 22RV1 cells treated with MLT (Figure S2D). Finally, we demonstrated that MLT promoted the CES1 expression in a dose‐dependent and an MT1‐dependent manner (Figures 2F and S2E).

### 2.3 CES1 was downregulated and predicted poor prognosis in PCa

**FIGURE 3:**
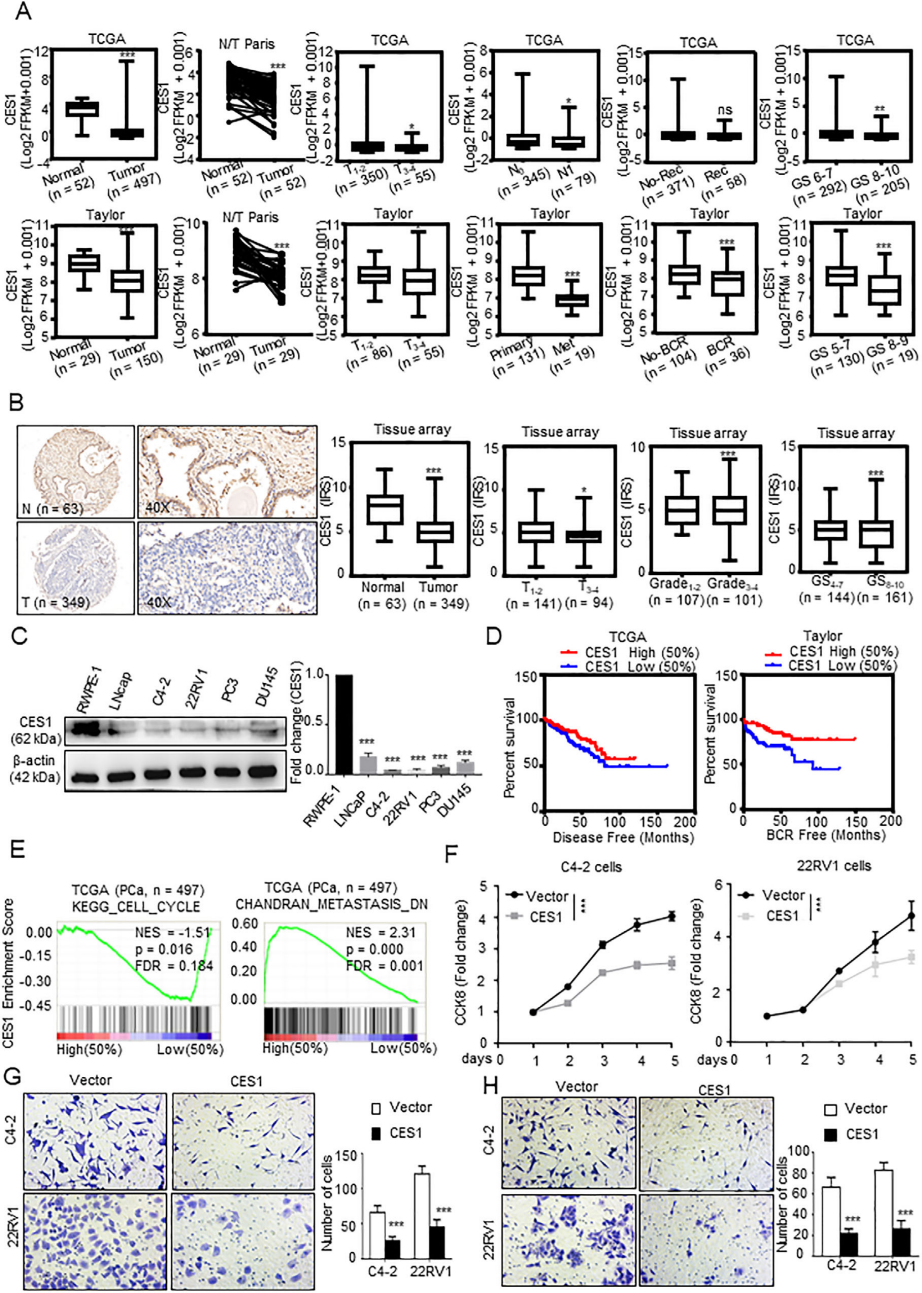
CES1 was downregulated and predicted poor prognosis in PCa. (A) Analysis based on the TCGA and Taylor databases; CES1 mRNA exhibited lower expression in the PCa tissues compared to that in the normal prostate tissues; low CES1 expression was positively correlated with tumor stage, metastasis, and Gleason score. Student's *t*‐test; *p < 0.05, **p < 0.01, and ***p < 0.001. (B) Representative images of IHC‐stained cells showing CES1 protein expression in the paraffin‐embedded prostate tissue arrays. CES1 expression in the PCa tissues was lower than that in the normal tissues, and lower CES1 expression was positively correlated with tumor stage, grade, and Gleason score. Student's *t*‐test; *p < 0.05; **p < 0.01; ***p < 0.001. (C) Detection of CES1 expression in PCa cell lines (LNCaP, C4‐2, 22RV1, DU145, and PC3 cells) and RWPE‐1 normal prostate epithelial cells by WB. Densitometry and statistical analysis. Representative images are shown. (D) Low levels of CES1 predicted poor DFS and BCR times in the TCGA PCa samples (*n* = 497) and Taylor database samples (*n* = 150). (E) GSEA of the correlation of the CES1 mRNA levels and cell cycle and metastasis‐related pathways in a TCGA PCa cohort. (F‐H) CCK‐8 and Transwell assays were performed to compare the effect of overexpressed CES1 (CES1) on cell activity, invasion, and migration compared to the control cells (vector). The results are presented as the mean ± SEM of three independent experiments. Student's *t*‐test; *p < 0.05, **p < 0.01, and ***p < 0.001

To investigate the effect of CES1 on PCa, we first analyzed CES1 expression in the independent PCa datasets (The Cancer Genome Atlas [TCGA], Taylor). The expression of CES1 was downregulated in PCa tissues compared to normal prostate tissues, and the high expression of CES1 is negatively correlated with tumor stage, metastasis, and Gleason score (Figure 3A). Receiver operating characteristic (ROC) curve showed that CES1 could be used as a diagnostic biomarker in PCa (Figure S3A). Multivariate analyses were carried out and demonstrating that CES1 was an independent prognostic marker in PCa (Figure S3B). Immunohistochemical (IHC) staining was carried out to determine the CES1 protein expression in the PCa specimens, and we found that the protein expression of CES1 was significantly lower in the PCa tissues compared with that in the normal prostate tissues, and high expression was negatively related to Gleason score and tumor stage (Figure 3B), indicating that CES1 is a key gene in PCa. Rabbit IgG (R‐IgG) as negative control (Figure S1F). The protein levels of CES1 in the PCa cell lines were lower than the level in the normal RWPE‐1 prostate epithelial cell line (Figure 3C). Furthermore, Kaplan‐Meier analysis of the data obtained from Taylor and TCGA databases indicated that patients with PCa who express low levels of CES1 (above the 50th percentile) had shorter BRFS (*p* = 0.0068) and DFS (*p* = 0.0282) than patients with PCa who express high levels of CES1 (below the 50th percentile) (Figures 3D and S3C). Therefore, we hypothesized that CES1 may be a tumor suppressor gene in PCa.

To prove our hypothesis that CES1 is a key gene in PCa, gene set enrichment analysis (GSEA) was executed to look for relative gene sets with CES1 expression in the PCa TCGA dataset. The results showed that pathways related to cell cycle and metastasis and pathways related to lipid accumulation and metabolism are associated with CES1 expression (Figures 3E and S3D). Then, using sgRNA (CRISPR‐Cas9) or lentivirus, we acquired 22RV1 and C4‐2 cell lines with knocked out or overexpressed CES1 (Figure S3E). Transwell and CCK‐8 assays were executed to examine whether CES1 is involved in the regulation of tumor growth and metastasis of PCa cells. C4‐2 and 22RV1 cells with stable overexpression of CES1 had significantly inhibited proliferation, migration, and invasion (Figures 3F‐3H).

### 2.4 MLT decreased lipid accumulation and cell activity in PCa cells by upregulating CES1 expression

**FIGURE 4:**
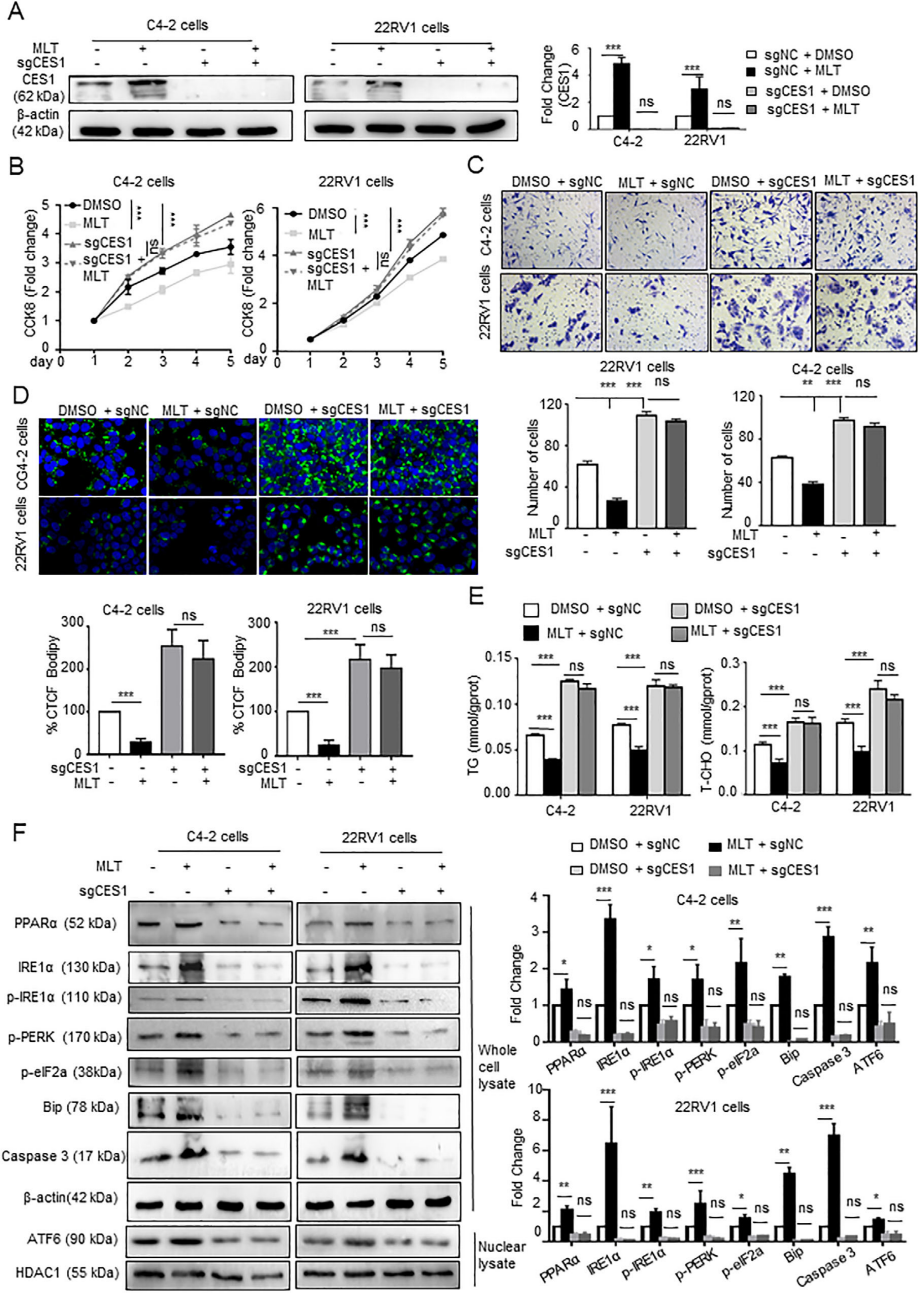
MLT inhibits lipid accumulation and PCa cell activity via CES1 expression. C4‐2 and 22RV1 cells with stable knockout of CES1 expression were generated and treated with DMSO or 1 mM MLT for 48 h. (A) Western blotting was performed to detect the CES1 expression in the groups. Densitometry and statistical analysis. Representative images are shown. (B and C) Cell viability and invasion under all conditions were determined using CCK‐8 and Transwell assays, respectively. (D) Knockout of CES1 abolished the inhibitory effect of melatonin on lipid accumulation. Representative images of BODIPY staining under all conditions. Quantification of the corrected total cell fluorescence (CTCF). (E) TG and T‐CHO contents were measured in all groups of cells. The graphs represent the means ± SEM. (F) Western blotting was performed to detect the expression of PPARa and ER stress marker genes in all groups. Densitometry and statistical analysis. Representative images are shown. A representative image of three independent experiments is shown. *p <  0.05, **p <  0.01, and ***p < 0.001. Abbreviation: ns, no significance

To investigate whether MLT affects PCa progression by CES1, 22RV1 and C4‐2 cells with CES1 knockout expression were treated with MLT. The results of Western blot indicated that MLT promotes the expression of CES1, and MLT negligibly promotes CES1 expression after inhibition of endogenous CES1 (Figure 4A). The results of CCK‐8 and Transwell assays suggested that CES1 knockout can almost completely abolish the repressions of MLT on the proliferation, invasion, and migration of C4‐2 and 22RV1 cells (Figures 4B, 4C, and S3F). Compared with the control cells, the cells treated with MLT showed lower fluorescence intensity, while the fluorescence intensity in the CES1‐knockout PCa cells with/without MLT treatment was similar, suggesting that CES1 knockout significantly abrogated the effects of MLT on the elimination of lipid deposits in PCa cells (Figure 4D). A similar result was found by TG and T‐CHO content assays (Figure 4E). These results indicate that MLT inhibits lipid accumulation and cell activity by upregulating CES1.

To investigate the mechanism of the effect of CES1 on lipid metabolism, a GSEA was performed; the results indicated that CES1 is positively related to the PPAR and ER lumen signaling pathway in the PCa TCGA database samples (Figure S3G). Previous studies have demonstrated that activation of the PPAR pathway leads to lipid degradation,^38^ and lipid droplet depletion increases ER stress and inhibits cell activity by mediating apoptosis.^39^ Combined RNA sequencing data indicated that treatment with MLT increases the expression of the PPARa pathway‐related genes CPT1C^40^ and ME1^41^ (Figure S3I). We hypothesized that MLT/CES1 mediates lipid depletion and apoptosis through the PPARa‐ER stress pathway. Our previous studies showed that melatonin reduced lipid drops accumulation in PCa cells by increasing the expression of the lipid metabolism‐related gene CES1. And a study by Bo Qiu et al from the University of Pennsylvania demonstrated that reduction of lipid drops induced ER stress through activation of UPR sensors PERK, IRE‐1α, and ATF6 signaling pathways.^39^ Therefore, we performed western blot assay to detect these signaling pathways. The results of Western blot demonstrated that MLT promotes the expression of PPARa, and the levels of phosphorylation of PERK, phosphorylation of eIF2a, and phosphorylation of IRE1a in MLT‐treatment cells are higher than the control cells, and ATF6 was significantly elevated in the nucleus after MLT treatment, while knocking out CES1 eliminated these effects (Figure 4F). These results indicated that MLT treatment activated the three pathways (PERK, IRE1a, and ATF6), which are involved in the UPR response.

Overall, these results indicate that MLT inhibits lipid accumulation and cell activity by regulating CES1 expression in PCa.

### 2.5 MLT inhibited CRPC progression and reversed enzalutamide resistance

**FIGURE 5:**
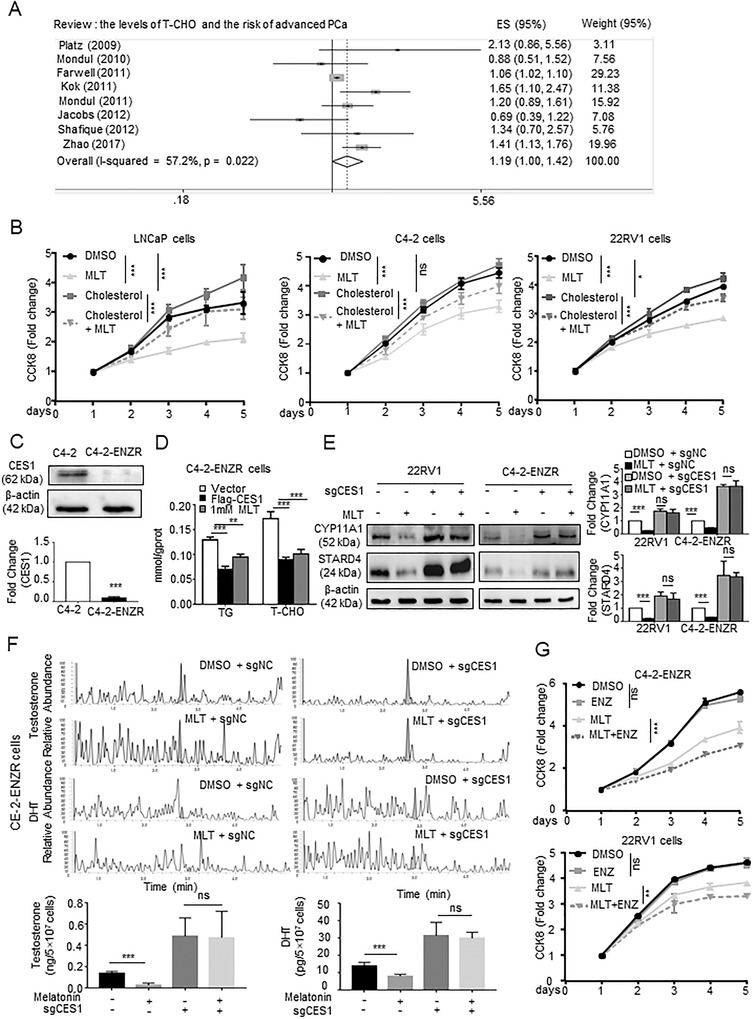
MLT inhibits CRPC progression and reverses enzalutamide resistance. (A) Meta‐analysis of the melatonin and T‐CHO levels in the blood. (B) Viability of the LNCaP, C4‐2, and 22RV1 cells treated with cholesterol (10 μM) and/or MLT (1 mM) was detected by CCK‐8 assay. (C) The CES1 expression in C4‐2‐ENZR cells compared with that in C4‐2 cells as determined by WB. Densitometry and statistical analysis. Representative images are shown. (D) TG and T‐CHO contents were measured in C4‐2‐ENZR cells treated with DMSO/MLT (1 mM) and in C4‐2‐ENZR cells with stable CES1 overexpression. The graphs represent the means ± SEM. (E) Western blotting was used to detect the effect of MLT treatment (1 mM, 48 h) on the expression of the proteins encoded by steroid hormone metabolism‐related genes (CYPA11A1 and STARD4) in enzalutamide‐resistant cell lines (C4‐2‐ENZR and 22RV1 cells). Densitometry and statistical analysis. Representative images are shown. (F) LC‐MS/MS was performed to evaluate the effect of MLT on the T (testosterone) and DHT (dihydrotestosterone) contents in C4‐2‐ENZR cells or in the cells with stable knockout of CES1 expression. The results represent the mean ± SEM of three independent experiments. (G) CCK‐8 assay was used to evaluate the viability of enzalutamide‐resistant cell lines (C4‐2‐ENZR and 22RV1 cells) treated with ENZ (20 μM) and/or MLT (1 mM). The data from a representative of three independent experiments are shown. *p <  0.05, **p <  0.01, and ***p <  0.001. Abbreviations: ns, no significance; RR, risk ratio; SMD, standard mean difference

Several studies have proved that the occurrence and progression of PCa, especially CRPC, are significantly related to the lipid metabolism.^10^ Higher levels of T‐CHO, TG, and LDL increase the risk for PCa, and lower HDL level is associated with a lower risk for PCa. The systematic review and meta‐analysis were carried out to demonstrate that high cholesterol levels are related to high incidence of advanced PCa (Figure 5A). These results indicated that cholesterol has a closely relationship with PCa progression. Cholesterol, which is stored in LDs in the form of cholesterol esters, is the raw material for androgen synthesis. Moreover, increased androgen synthesis in tumor cells is an important factor in the progression of PCa to CRPC and enzalutamide resistance. Since MLT‐mediated CES1 expression inhibits lipid accumulation in PCa cells and decreases intracellular cholesterol content (Figures 2D and 4E), we hypothesized that MLT/CES1 reduces cholesterol, leading to the interference of PCa progression.

To test this hypothesis, we initially performed a GSEA with TCGA PCa datasets and found that CES1 is associated with a set of positively regulated cholesterol efflux genes and a set of metabolism‐related steroid hormones genes (Figure S3J). Then, CCK‐8 assays were performed to assess the effects of cholesterol and/or melatonin on PCa cells at various stages. LNCaP cells are sensitive to androgen; C4‐2 cells constitute a CRPC cell line derived from LNCaP cells and express functional endogenous AR^42^; 22RV1 cells constitute a CRPC and ENZ‐resistant cell line that expresses AR‐V7.^43^ Cholesterol treatment promoted the proliferation of PCa cells, and the LNCaP cells cultured without androgen were more sensitive to cholesterol treatment than the CRPC cell lines (C4‐2 and 22RV1 cells) suggesting that the effect of cholesterol on PCa progression is associated with androgen; MLT inhibited the proliferation of these cells, and cholesterol supplementation partially reversed MLT‐mediated inhibition (Figure 5B).

Moreover, we generated a C4‐2‐ENZR cell model (C4‐2‐enzalutamide resistance cell) to test whether MLT/CES1 influences enzalutamide resistance. We found that the expression of CES1 in the C4‐2‐ENZR cells is lower than that in the C4‐2 cells (Figure 5C); MLT treatment had the opposite effect (Figure S3K). MLT or overexpressed CES1 reduced the cholesterol and TG contents in C4‐2‐ENZR cells (Figures 5E and S3H). Considering the RNA sequencing data, we determined that treatment with MLT significantly decreased the expression levels of androgen synthesis‐related genes CYP11A1^44^ and STARD4^45^ (Figure S3I). The Western blot data demonstrated that the levels of the proteins encoded by the androgen synthesis‐related genes CYP11A1 and STARD4 were significantly lower in the MLT‐treated C4‐2‐ENZR and 22RV1 cells than those in the control cells (Figure 5E), indicating that MLT influences intratumoral androgen synthesis in enzalutamide‐resistant CRPC. Thus, we performed an LC/MS (liquid chromatography‐mass spectrometry) analysis to evaluate the levels of testosterone (T) and dihydrotestosterone (DHT) in tumor cells. The results showed that MLT seriously decreased the contents of T and DHT in the C4‐2‐ENZR cells and had a negligible effect in the absence of endogenous CES1, demonstrating that MLT inhibited intratumoral androgen synthesis though CES1 (Figure 5F).

Finally, we conducted CCK‐8 assays and found that the C4‐2‐ENZR and 22RV1 cells were resistant to ENZ treatment and that treated‐MLT restored the sensitivity of ENZ treatment in 22RV1 and C4‐2‐ENZR cells (Figure 5G).

### 2.6 MLT‐mediated epigenetic modification upregulated CES1 expression

**FIGURE 6:**
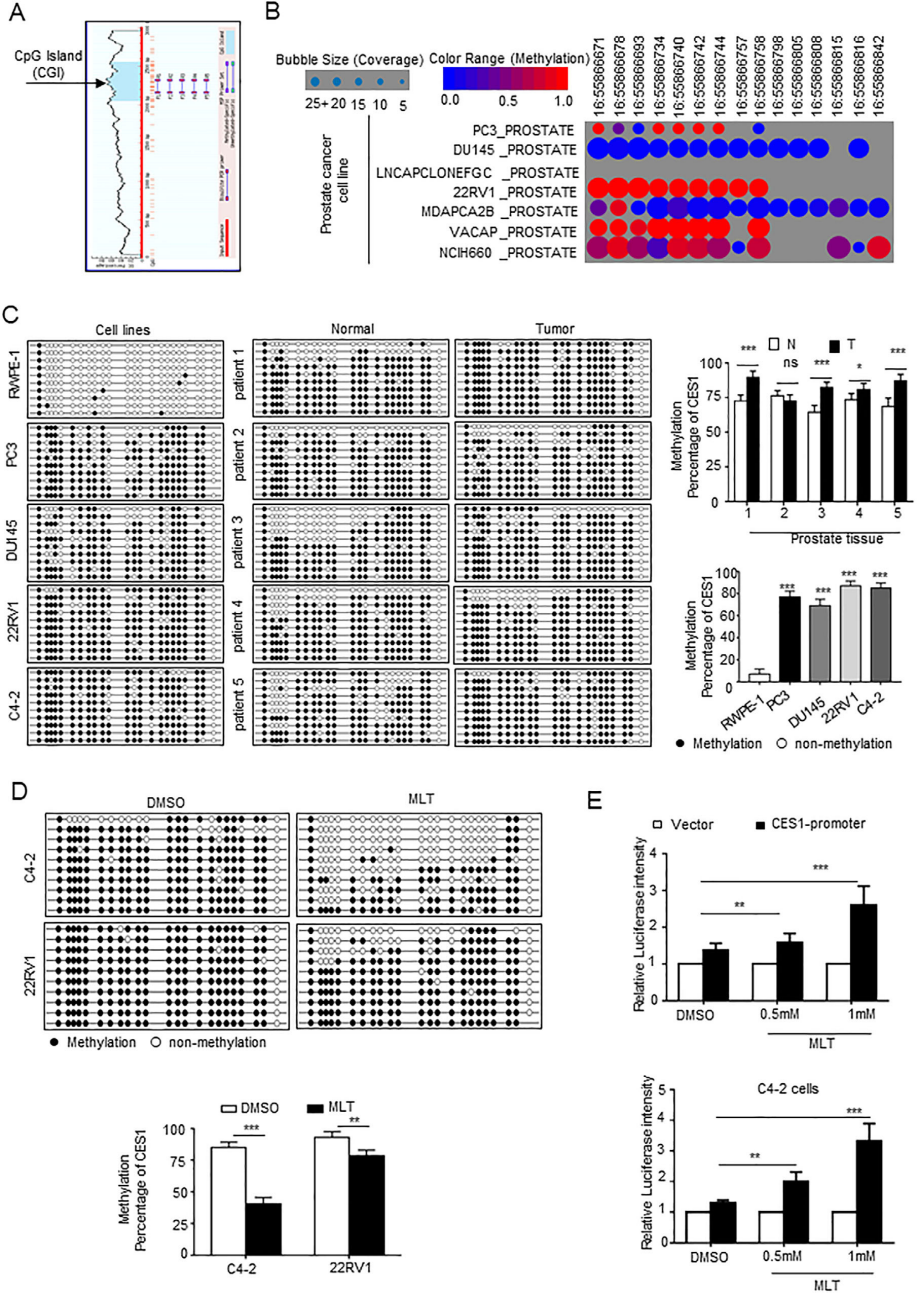
MLT‐mediated epigenetic modification upregulates CES1 expression. (A) Prediction of the CpG islands (CGIs) in CES1 promoter regions using MethPrimer. (B) The publicly available database Cancer Cell Line Encyclopedia (CCLE) was used in bioinformatics analyses to evaluate the methylation status of the CES1 promoter in PCa cell lines. (C) Detection of the methylation status of the CES1 promoter in PCa cells (PC3, C4‐2, 22RV1, and DU145), normal prostate epithelial cells (RWPE‐1), five paired PCa tissue and adjacent normal prostate tissue samples using BSP sequencing. Then statistical analysis. (D) Using BSP assays, detect and statistical analysis for the CGI methylation status of the CES1 promoter in C4‐2 and 22RV1 cells treated with 1 mM MLT (1 mM, 48 h) compared to that in the control (DMSO treatment). (E) Luciferase reporter assays were performed after cotransfection of the pGMLR‐CMV luciferase reporter and the CES1 promoter or blank vector plasmids in 22RV1 and C4‐2 cells treated with various concentrations of MLT

Abnormal DNA methylation is an important epigenetic alteration in cancer. In tumors, 5′‐ UTRs and CpG islands (CGIs) are hypermethylated relative to prostate tissue.^46^ The results of the analysis for the CES1 genomic DNA sequence in the 2‐kilobase promoter regions indicated that there are CGIs in these regions (Figure 6A). Besides, we used the Cancer Cell Line Encyclopedia database to evaluate the methylation status of the CES1 promoter in several PCa cell lines and found that the methylated CES1 promoter is relatively enriched in CRPC cells (Figure 6B). The results of bisulfite sequencing PCR (BSP) indicated there were noticeable hypermethylation of the CES1 promoter in PCa cells compared with that in normal prostate epithelial cells, while similar result in PCa tissues compared with that in paired normal prostate tissues (Figures 6C and S4A).

Considering the downregulation of CES1 in PCa cells and tissues, the results indicate that DNA methylation may be the key regulatory factor of CES1 expression. To determine the mechanism by which CES1 expression is upregulated in PCa by MLT treatment, BSP was performed to assess the changes in the methylation level of the CES1 promoter after MLT treatment. As shown in Figure 6D, MLT treatment reduced CGIs methylation of the CES1 promoter. The results of the luciferase reporter assays showed that the luciferase activity of the cells transfected with a vector containing the CES1 promoter was promoted by the MLT treatment of the C4‐2 and 22RV1 cells in a dose‐dependent manner (Figure 6E). Overall, these results indicate that MLT‐mediated demethylation upregulates CES1 expression.

### 2.7 MLT regulated methylation of CES1 promoter though SIRT1‐mediated DNMT1 deacetylation

**FIGURE 7:**
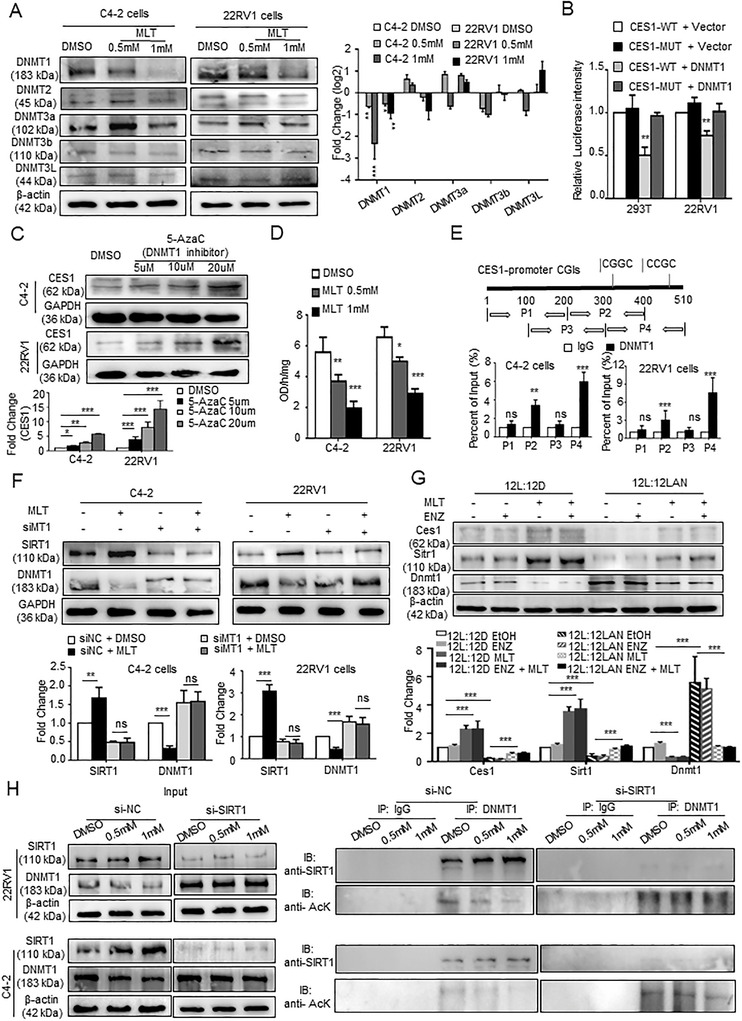
MLT regulated methylation of CES1 promoter though SIRT1‐mediated DNMT1 deacetylation. (A) WB was used to assay DNMT (DNMT1, DNMT2, DNMT3a, DNMT3b, and DNMT3L expression after treatment with various concentrations of MLT. Densitometry and statistical analysis. Representative images are shown. (B) Luciferase reporter assays were performed after co‐transfection of 22RV1 and 293T cells with a pGMLR‐CMV luciferase reporter and CES1 promoter (CES1‐WT) vector or a blank vector (CES1‐MUT) plasmid with a DNMT1 overexpression or blank vector plasmid. (C) CES1 protein levels were determined by Western blotting in cells treated with DNMT1 inhibitor 5‐aza‐dC (0 μM, 5 μM, 10 μM, and 20 μM) for 4 days. Densitometry and statistical analysis. Representative images are shown. (D) DNMT activity assay kits were used to assay the DNMT activity in 22RV1 and C4‐2 cells treated with various concentrations of MLT. (E) ChIP‐PCR assays were performed to detect direct binding of DNMT1 to the CES1 promoter regions in 22RV1 and C4‐2 cells. (F) WB was used to detect SIRT and DNMT1 protein expression levels in the C4‐2 and 22RV1 cells treated with MLT (1 mM) 48 h after transfection with siMT1. Densitometry and statistical analysis. Representative images are shown. (G) WB was used to detect the expression of Ces1, Sirt1, and Dnmt1 in the animal CRPC models with a circadian rhythm disorder. Densitometry and statistical analysis. Representative images are shown. (H) After transfection with siSIRT1 or siNC, 22RV1 and C4‐2 cells were treated with various concentrations of MLT. The cell lysate was immunoprecipitated with anti‐DNMT1 antibody, and the precipitated proteins were assayed by Western blotting with anti‐SIRT1 and anti‐acetyl‐lysine (anti‐AcK) antibodies. Representative images are shown. The data from a representative of three independent experiments are shown. *p < 0.05, **p < 0.01, and ***p < 0.001. Abbreviation: ns, no significance

In mammals, genomic methylation status is established by DNA methyltransferases (DNMTs), including DNMT1, DNMT2, DNMT3a, DNMT3b, and DNMT3L.^47^ Western blot was carried out to determine whether MLT influences DNA methylation of the CES1 promoter via DNMTs, and the results indicated that the level of DNMT1 was significantly and consistently decreased in a dose‐dependent manner, in the 22RV1 and C4‐2 cells treated with MLT, compared to the control cells (Figure 7A). Luciferase reporter assays demonstrated that the luciferase activity of the cells transfected with a vector containing the CES1 promoter was inhibited by DNMT1 overexpression in 293T and 22RV1 cells (Figure 7B). Note that 5‐azacytidine (5‐AzaC), an inhibitor targeting DNMT1, upregulated CES1 protein expression in a dose‐dependent manner (Figure 7C). Moreover, MLT decreased the activity of DNMT1 in a dose‐dependent manner (Figure 7D). Then, chromatin immunoprecipitation (ChIP) assays were executed. A strong enrichment between the CGIs of CES1 promoter and anti‐DNMT1 antibody was observed in the 22RV1 and C4‐2 cells, while MLT decreased this enrichment in dose‐dependent (Figures 7E and S4B). These results indicate that MLT suppresses DNA methylation of CES1 by inhibiting DNMT1 binding to CGIs.

Several studies have demonstrated that SIRT1 deacetylates the DNMT1 protein and alters its activity.^48^ Numerous findings have reported an increase in the activity of SIRT1 after MLT treatment.^49^ We hypothesized that MLT regulates DNMT1 by SIRT1‐mediated deacetylation. The results of the Western blot showed that MLT promoted SIRT expression and decreased the DNMT1 protein level in an MT1‐dependent manner (Figure 7F). Moreover, LAN‐mediated circadian rhythm disorder downregulated the expression of Ces1 and Sirt1, upregulated the expression of Dnmt1, while MLT supplementation eliminated the effects in the mouse tumor tissues (Figure 7G). We constructed siRNA for SIRT1 (Figure S4C). The results of the coimmunoprecipitation (co‐IP) assay showed that MLT treatment increased SIRT1 binding to DNMT1 and induced DNMT1 deacetylation in dose‐dependent manner, and SIRT1 knockout eliminated the effect of melatonin on the DNMT1 expression (Figures 7H, S4D, and S4E).

Overall, these results indicate that MLT reduces the methylation level of the CES1 promoter by upregulating SIRT‐mediated DNMT1 deacetylation.

### 2.8 MLT inhibited tumor growth and reversed enzalutamide resistance of CPRC by mediating CES1 expression in vivo

**FIGURE 8:**
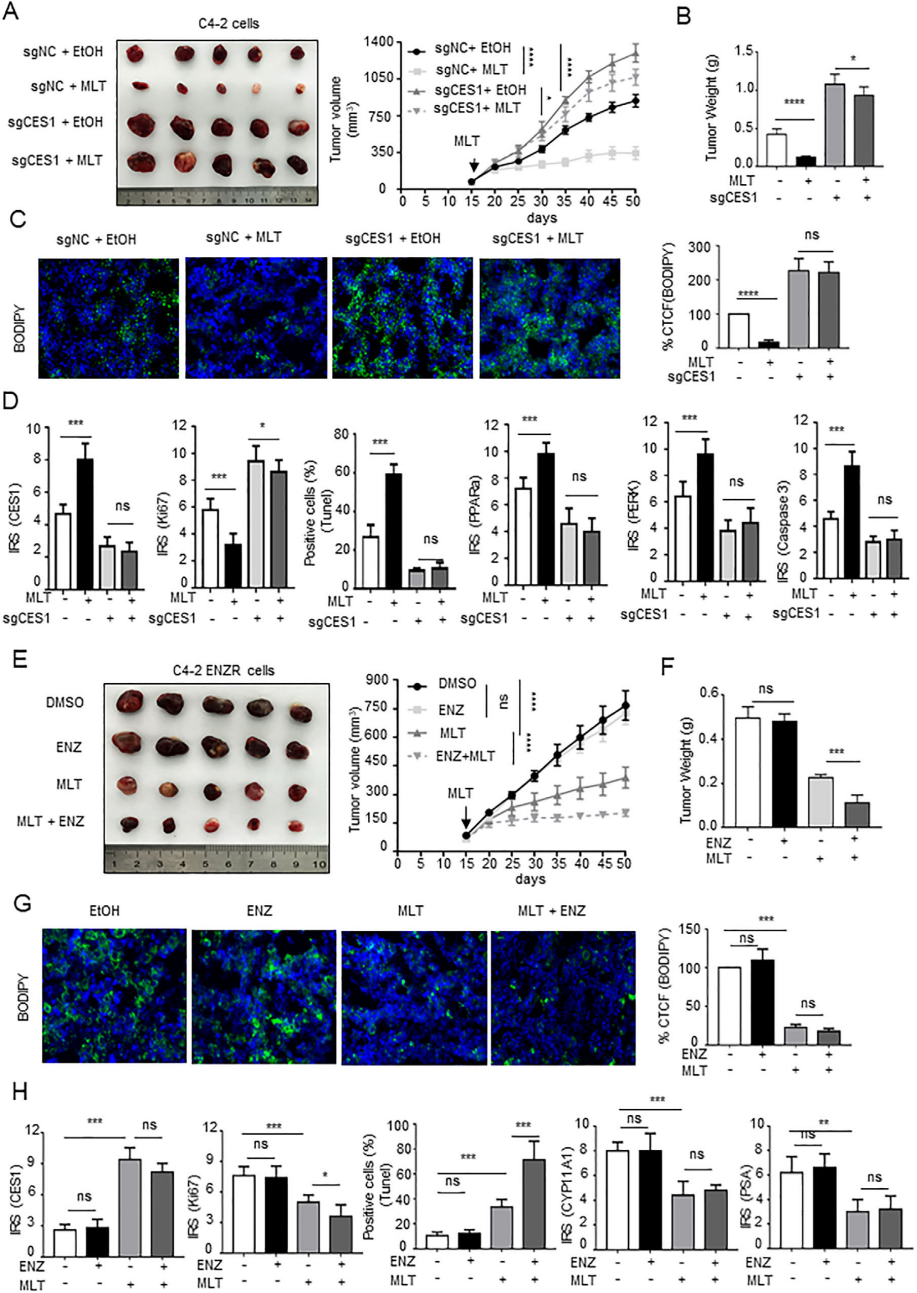
MLT inhibits tumor growth and reverses enzalutamide resistance of CPRC via CES1 expression in vivo. (A and B) NCG mice bearing C4‐2 xenograft tumors with stable knockout of CES1 expression were treated with vehicle control (EtOH) or MLT (200 mg/kg, intragastric administration) for approximately 7 weeks. Tumor volumes were measured every 5 days (*n* = 5 per group). Tumors were weighed after the resection. The graphs show the means ± SEM. One‐way ANOVA followed by Tukey's multiple comparison test, *α* = 0.05; *p < 0.05, **p < 0.01, and ***p < 0.001. (C) BODIPY staining assays were performed to detect intracellular lipids in the C4‐2 cell xenograft tumor tissues. Representative images are shown. Quantified the corrected total cell fluorescence (CTCF) of BODIPY stained. (D) Statistical analysis of the IRS scores of IHC stained C4‐2 cell xenograft tumor tissues. (E and F) NCG mice bearing C4‐2‐ENZR cell xenograft tumors were treated with vehicle control (EtOH) or MLT (200 mg/kg, intragastric administration) or/and ENZ (10 mg/kg, intragastric administration) as described in “Materials and Methods.” Tumor volumes were measured every 5 days (*n* = 5 per group). Tumors were weighed after the resection. The graphs show the means ± SEM. One‐way ANOVA followed by Tukey's multiple comparison test, *α* = 0.05; *p < 0.05; **p < 0.01; ***p < 0.001. (G) BODIPY staining assays were performed to detect intracellular lipids in the C4‐2‐ENZR cell xenograft tumor tissues. Representative images are shown. Quantified of the corrected total cell fluorescence (CTCF) of BODIPY staining. (H) Statistical analysis of the IRS scores of IHC‐stained C4‐2‐ENZR cell xenograft tumor tissues. Abbreviation: ns, no significance

To study the role of MLT/CES1 in vivo, C4‐2 cells were lentivirally transduced to knockout CES1 expression, implanted subcutaneously in NCG mice. C4‐2‐cell xenograft tumor treated with MLT (200 mg/kg, intragastric administration) was significantly smaller with a reduced growth rate, and weight compared with tumors treated with a vehicle (EtOH), and C4‐2 tumor with stably knocked out CES1 expression showed an opposite pattern of tumor growth compared with that of the vector‐transduced tumor. Knockout of CES1 reversed the effect of MLT on tumor growth (Figures 8A and 8B). Notably, BODIPY staining revealed sparse lipid deposition in the MLT‐treated tumor compared to that in the control tumor; knockout of CES1 expression increased lipid accumulation and reversed the effect of melatonin (Figure 8C). Moreover, the results of the IHC and IF assays indicated that melatonin upregulated the levels of CES1, PPARa, ER stress markers, and caspase‐3‐mediated apoptosis, and CES1 knockout abolished these MLT‐induced effects, R‐IgG as negative control (Figures 8D and S5A). Overall, these results indicate that MLT inhibits tumor growth by upregulating the CES1 expression in vivo.

Considering these results, we studied the MLT‐mediated reversal of enzalutamide resistance in vivo. C4‐2‐ENZR cells were implanted subcutaneously in NCG mice. The results indicate that tumors treated with MLT alone reduced tumor size, growth rate, and weight compared with those in the control group; however, the size, growth rate, and weight of the tumors treated with ENZ alone have no significant differences from those in the control group; those of the tumors in mice fed MLT and ENZ were the most obviously decreased (Figures 8E and 8F), indicating that MLT increased the sensitivity of enzalutamide‐resistant CRPC to ENZ. BODIPY staining and IF and IHC assays were performed with the xenograft tumors. MLT treatment decreased lipid accumulation, upregulated the levels of CES1, inhibited cell proliferation, decreased androgen synthesis, and increased the apoptosis rate (Figures 8G, 8H, and S5B). Moreover, the results of IHC demonstrated that LAN‐induced circadian rhythm disorder downregulated the expression of Ces1, Ppara, Perk, caspase‐3, Cyp11a1, and Psa, R‐IgG as negative control. MLT treatment abolished these effects in the PCa tissues of the C57BL/6 mice (Figures S6A and S6B). Thus, the data indicate that MLT reverses enzalutamide resistance of CPRC.

### 2.9 Ces1‐knockout mice confirmed the role of endogenous *Ces1*

**FIGURE 9:**
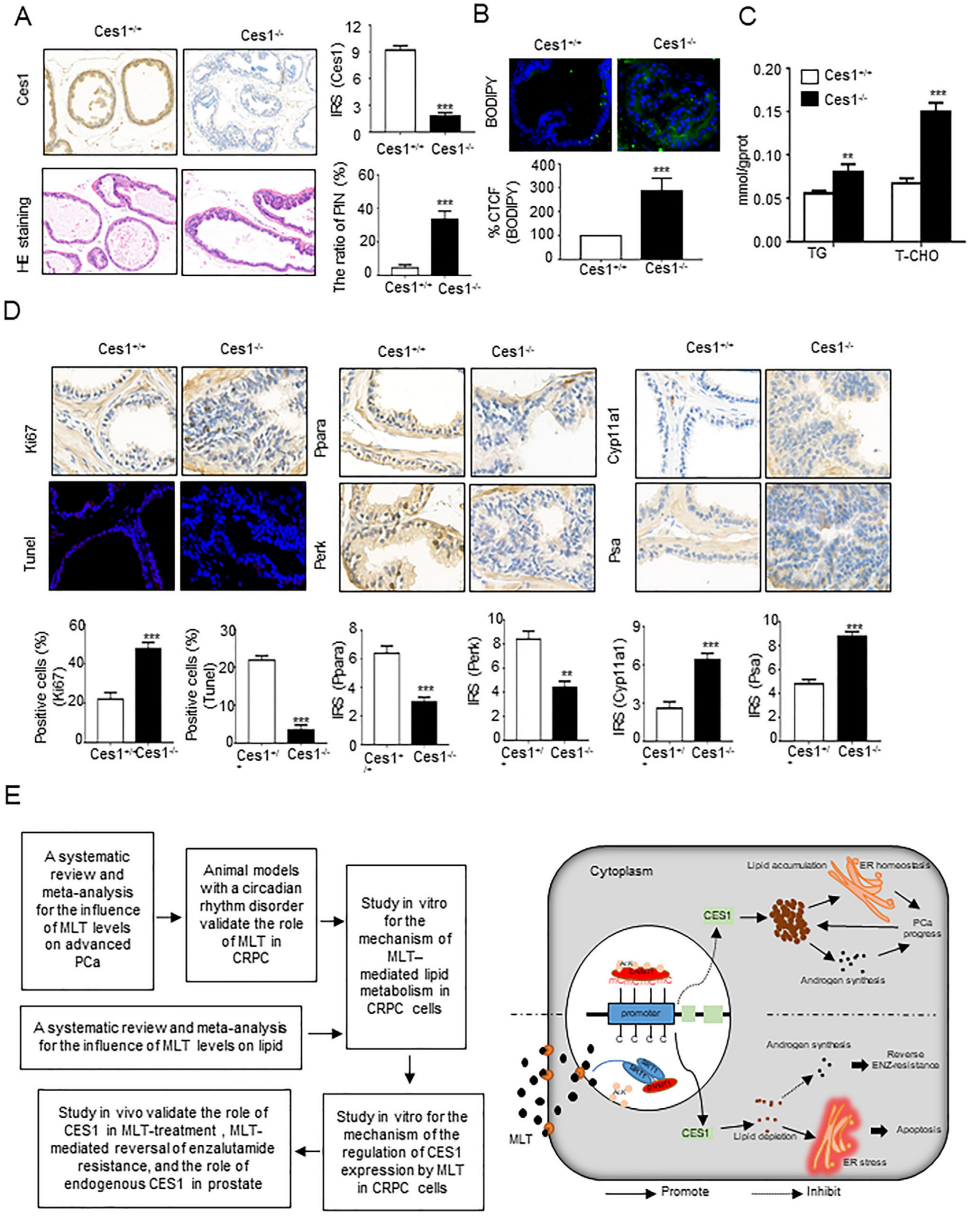
Confirmation of the role of endogenous *Ces1* using CRISPR/Cas9 Ces1‐knockout mice. (A) The results of IHC and HE staining demonstrate the loss of endogenous Ces1 and prostatic intraepithelial neoplasia (PIN) in the anterior prostate of transgenic mice (*n* = 10 mice per group). Statistical analysis. Representative images are shown. (B and C) Quantification of the corrected total cell fluorescence (CTCF) of BODIPY staining and detection of TG and T‐CHO contents in all groups of prostate tissues were used for generating indicators of intracellular lipids. The graphs show the means ± SEM (*n* = 10 mice per group), Student's *t*‐test; *p < 0.05, **p < 0.01, and ***p < 0.001. (D) IHC and TUNEL assays were performed to detect the levels of Ces1, Ki67, Ppara, Perk, caspase‐3, Cyp11a11, and Psa in the Ces1^−/−^ and Ces1^+/+^ prostate tissues and the apoptosis rate of the Ces1^−/−^ and Ces1^+/+^ prostate tissue cells. Representative images are shown. Statistical analysis of the IRS scores of IHC staining of CES1‐/‐ or Ces1+/+ prostate tissues. The graphs show the means ± SEM (*n* = 10 mice per group), Student's *t*‐test; *p < 0.05, **p < 0.01, and ***p < 0.001. (E) A workflow of the entire study is shown. And, proposed model illustrating the protective function of MLT‐mediated lipid depletion in PCa progression

Ces1‐knockout mice (Ces1^−/−^) were generated to investigate the endogenous CES1's role in vivo. IHC and HE staining assays were performed and demonstrated that loss of CES1 induced the higher ratio of prostatic intraepithelial neoplasia (PIN) in the anterior prostates (Figure 9A), suggesting that the absence of CES1 mediated the transformation of normal prostate tissue into tumorous tissues. R‐IgG as negative control (Figure S6C). As quantitative indicators of lipid accumulation, the contents of TG and cholesterol measurement and BODIPY staining assays were carried out in the prostate tissues. As shown in Figures 9B and 9C, lipid accumulation was increased in the Ces1^−/−^ prostate tissues compared to the control mouse prostate tissues. Moreover, as shown in Figure 9D, the extent of Ki67 staining was markedly increased, while the extent of Perk and caspase‐3 staining was significantly reduced in the Ces1^−/−^ prostate tissues, indicating that Ces1 repressed cell proliferation and induced ER stress‐mediated apoptosis. The percentage of apoptotic cells was decreased in the Ces1^−/−^ prostate tissues, suggesting that the lack of endogenous CES1 facilitated cell survival; the Ces1^−/−^ prostate tissues had lower Ppara staining intensity comparing to the control group and higher intensity of Cyp11a1 and Psa staining, indicating that Ces1 mediated LD elimination and reduced androgen synthesis in the prostate cells.

Figure 9E shows the schematic diagram of this process. In an MT1‐dependent manner, MLT regulated epigenetic modification of CES1 through SIRT1‐mediated DNMT1 deacetylation. MLT‐mediated CES1 expression induced lipid depletion, leading to ER stress‐related apoptosis and blocking the intratumoral androgen synthesis that results in the inhibition of PCa progression and reversal of the enzalutamide resistance.

## 3 DISCUSSION

Endocrine therapy, repressing the androgen/AR signaling pathway, has always been the first‐line treatment for advanced PCa. However, continuous inhibition of androgen/AR signaling pathway does not prevent PCa progression. Although androgen production from the testicles and adrenal glands is blocked, androgen synthesis is increased in PCa cells.^12^, ^13^ Besides, with a high sensitivity to low levels of androgens, AR variants drive disease progression. AR‐V7, the most common AR splice variant, encodes a truncated AR protein that lacks the ligand‐binding domain, which is the target of enzalutamide and abiraterone, but still has constitutive activity as a transcription factor.^50^, ^51^ When PCa progresses to CRPC, enzalutamide or abiraterone (new endocrine therapy [NET]) was used as a clinical treatment to further inhibit AR pathway, but the tumor still cannot be cured eventually. It is urgent to find new therapeutic perspectives.^52^ Recent studies have shown that AR variation‐mediated cholesterol synthesis, and lipid deposits contribute to CRPC progression and NET resistance. As a precursor of steroid synthesis, cholesterol increases androgen production in tumor cells and is upregulated in NET‐resistant CRPC cells. Increased androgen synthesis in tumors would activate androgen pathways and promote CRPC progression. Based on the above analysis, we concluded that the positive feedback loop formed by AR activation, lipid/cholesterol, and androgen in tumors promotes CRPC progression and NET resistance, and intratumoral lipid/cholesterol accumulation is a key factor in this process. In the current studies, we found that MLT decreased the lipid/cholesterol accumulation to repress CRPC progression and reversed enzalutamide resistance, and this effect is mediated by the epigenetic modification of CES1.

CES1 is a member of the serine hydrolase superfamily primarily located in the endoplasmic reticulum in many tissues and plays a key role in endobiotic metabolism, especially in the hydrolysis of cholesteryl esters and TGs.^29^ Compared with endocrine therapy that eventually fails and leads to lipid accumulation, MLT treatment or restoration of CES1 expression is to repress lipid/cholesterol accumulation, promote tumor cell slimming in tumors and reduce de novo intratumoral androgen synthesis. The MLT treatment combined with NET will enhance the therapeutic effects and offer a promising new treatment modality against PCa.

Previous studies have demonstrated that lipid storage promotes ER homeostasis and significantly increases tumor cell proliferation and viability.^39^ Stimulation by internal and external environmental stress factors, such as hypoxia, induces alterations in the homeostasis of endoplasmic reticulum known as ER stress. ER stress is thought to restore the ER homeostasis; however, persistent and irremediable ER stress can result in cell death.^39^, ^53^ In melanoma, p‐PERK and eIF2a levels are the critical initiators of ER stress and are significantly increased by treatment with melatonin.^54^ The results of the present study indicate that MLT decreases lipid/cholesterol accumulation and induces ER stress to inhibit PCa progression. Several enzymes, CYP17A1, CYP11A1, HSD3B, STARD4, and AKR1C3, are involved in the biosynthesis of intratumoral androgens.^14^, ^44^, ^45^, ^55^ Here, we find that MLT downregulates the expression of CYP11A1 and STARD4 in PCa cells, suggesting that MLT represses the intracrine androgen biosynthesis not only by reducing lipid/cholesterol accumulation, but also by inhibiting the enzymes involved in androgen synthesis.

Many studies, including randomized controlled trial and clinical trial dealing with MLT and breast cancer, have reported that oral melatonin treatment significantly improves the quality of life of patients, not only by improving the cognitive function, sleep quality, and other mental states of patients,^56^, ^57^, ^58^, ^59^, ^60^ but also by inhibiting the adverse reactions of radiotherapy and chemotherapy, and the progression of tumors through a variety of ways.^61^, ^62^, ^63^, ^64^, ^65^, ^66^, ^67^ MLT, as a multifunctional indoleamine, plays a role in lipid metabolism and anticancer activities. Long‐term disruption of circadian rhythms with decreased MLT secretion modestly increased risk for many types of cancer. But the role and mechanism of melatonin in CRPC, whether MLT represses PCa progression thought mediating lipid metabolism, are not quite clear, especially in enzalutamide‐resistant CPRC. The results of the present study indicate that MLT represses CRPC progression and reverses enzalutamide resistance by inhibiting lipid accumulation.

MLT have multiple functions in cancers. In this research, MLT regulates many key molecules in lipid metabolism and tumor microenvironment. Combined with the comprehensive phenotype of MLT and PCa, lipid metabolism plays an important role in MLT‐mediated tumor inhibition process. Meanwhile, MLT is a natural antioxidant with immune and inflammation‐regulated properties though cell cytokine; therefore, we believe that MLT also inhibits PCa progression through the bypass pathway. Discovering the bypass, perfecting the mechanism of MLT in CRPC progression will improve the overall treatment efficiency. Besides, lipid metabolism is a complex process, not determined by a single gene. In Top30 DEGs of MLT treatment, there are eight genes related to lipid metabolism, although not changed as significantly as CES1. SLC7A10 and SLC38A4 act as the amino acid transporter, regulating lipid storage in adipocyte.^33^, ^68^ SLC22A18 regulating the IGFBP1 expression increased the supply of intracellular FFAs from TG‐rich lipid droplets.^69^ ALDH1A2 and DHRSX are enzymes that cause lipid peroxidation.^27^, ^70^ ACSBG1is an acyl‐CoA synthetases that activate fatty acids to their CoA derivatives play a central role in fatty acid metabolism.^71^ Zic1 a classical lipid browning marker.^72^ The transcription factor HNF4A regulates expression of genes required for fatty acid oxidation.^28^ MLT may also regulate lipid metabolism thought these pathways. We also found that CES1 knockout inhibition of MLT on lipid metabolism was incomplete in functional experiments. Whether MLT has bypass or other mechanisms to regulate lipid metabolism genes will become the focus of our exploration in the next stage.

In summary, our results demonstrate that MLT mediated epigenetic modification of CES1, inhibits lipid accumulation to induce ER stress‐mediated apoptosis, suppress intracrine androgen biosynthesis, ultimately repress CRPC progression, and reverse enzalutamide resistance. This study is the first to report a direct role of melatonin in lipid reduction, mediation of CRPC progression, and enzalutamide resistance of CRPC. These findings provide novel preclinical and clinical information about the role of melatonin in advanced PCa and highlight the importance of second‐generation antiandrogen therapy combined with MLT treatment for advanced PCa.

## 4 MATERIALS AND METHODS

### 4.1 Cell culture and reagents

Human PCa cell lines LNCaP, C4‐2, 22RV1, PC3, and DU145 and mouse‐derived PCa cell line Rm‐1 were cultured in RPMI 1640 medium supplemented with 10% fetal bovine serum (FBS) (Gibco, USA) at 37°C and 5% CO_2_. RWPE‐1 cells were incubated in K‐SMF medium (Gibco) at 37°C and 5% CO_2_. Note that 293T cells were incubated in DMEM supplemented with 10% FBS at 37°C and 5% CO_2_. All cell lines were obtained from the American Type Culture Collection (ATCC).

Lentiviruses for the expression or knockout of CES1 and the corresponding control vectors were purchased from GeneChem. siRNAs for SIRT or MTNR1A (MT1) and control siRNA were obtained from RiboBio. All transfection steps were performed according to the manufacturers’ instructions. PCa cells were treated with various concentrations (0, 0.5, and 1 mM) of melatonin (Sigma) for 48 h according to previous studies.^37^

### 4.2 Human samples

Paired adjacent noncancer and cancer paraffin‐embedded tissue arrays for IHC staining were bought from Shanghai Outdo Biotech (HProA150CS03), Xi'an Alena Bio (DC‐Pro11027 and DC‐Pro11036), Xi'an ZK Bioaitech (M821601), and Suzhou CCELL (20190522R80). Detailed clinical information (pathological and clinical stage diagnoses which were given by pathologist) was directly provided by the companies. The fresh PCA tissues and adjacent normal tissues used for BSP (bisulfite genomic sequence polymerase chain reaction [PCR]) were obtained from the Department of Urology, Union Hospital, Tongji Medical College, Wuhan, China; the samples were frozen and stored in liquid nitrogen. Prior informed consent was obtained from each patient, and the study was approved by the Institutional Research Ethics Committee.

### 4.3 Animals and melatonin administration

All animal procedures have been approved by the Animal Care and Use Committee of Tongji Medical College of Huazhong University of Science and Technology.

Male C57BL/6 mice, 4‐ to 5‐week‐old, which had been surgically castrated, were bought from the Vital River Laboratory Animal Technology Co. Ltd. (Beijing, China). The animals were individually maintained in an isolated room at 25°C, with humidity of 60 ± 10% and a diurnal lighting schedule controlled by an electronic timer according to the experimental design (12 h light:12 h dark; 12L:12D, 200–300 lux; lights on 06:00 h, off at 18:00 h). One week before the tumor was implanted, one‐half of the animals were changed to continuous light (12L:12LAN, 12 h light:12 h light at night), as described previously. Rm‐1 cells in PBS (2 × 10^6^) were subcutaneously inoculated into each mouse. When the tumor reached approximately 0.5 cm, MLT, ENZ, or Vehicle (ETOH) treatment was initiated. MLT (Solarbio, Beijing, China) was dissolved in 0.02% ethanol to a final concentration of 60 mg/ml. As described previously, the stock solution was diluted to 0.1 mg/ml by adding drinking water in lightproof bottles and was administered to the mice from 18:00 to 06:00 to simulate MLT with high normal nocturnal physiological levels. The animals treated with intragastrically were administered 10 mg/kg ENZ (MedChemExpress) daily for approximately 7 weeks as described in a previous study. The present study included eight groups of five mice each: group I (12L:12D, EtOH), group II (12L:12D, ENZ), group III (12L:12D, MLT), group IV (12L:12D, MLT + ENZ), group V (12L:12LAN, EtOH), group VI (12L:12LAN, ENZ), group VII (12L:12LAN, MLT), and group VIII (12L:12LAN, ENZ + MLT). Tumor size was measured every 5 days using a digital caliper. After 55 days, the mice were euthanized, and the tumors were removed, measured, stored, and fixed in phosphate‐buffered formalin for future study.

Male NCG mice (4‐ to 5‐week‐old), purchased from GemPharmatech., Ltd. (Nanjing, China), were surgically castrated prior to the use in the experiments in a subcutaneous xenograft model investigating the role of MLT/CES1 or MLT‐induced reversal of enzalutamide resistance in vivo. Cells (5 × 10^6^) in a 1:1 mixture of PBS:Matrigel (BD, USA) were subcutaneously inoculated into each mouse. The animals were treated with MLT (200 mg/kg by intragastric administration). Measure the tumor size every 5 days. The mice were sacrificed on day 55 after cell implantation, and the tumors were excised, measured, stored, and fixed in phosphate‐buffered formalin for future study.

Ces1‐knockout mice (Ces1^−/−^) constructed by the CRISP/Cas9 technique were purchased from Cyagen Bio‐Technique Co., Ltd. (Suzhou, Jiangsu, China).

### 4.4 Cell proliferation assays

Five thousand cells/well were plated in 96‐well plates for cell proliferation assay and cultured overnight at 5% CO_2_ and 37°C. According to the Cell Counting Kit‐8 (CCK‐8) manufacturer's instructions, cell proliferation rate was determined. All experiments were carried out in triplicate.

### 4.5 Transwell assays

To assess the migration and invasion, cells were cultured to 80% fusion in six‐well plates, then incubated in serum‐free medium for 24 h. The Transwell chamber used for invasion tests was pre‐coated with Matrigel (BD Biosciences, San Jose, CA, USA). The cells (2 × 10^4^) were cultured in the top chamber of an insert, and medium containing 10% FBS was added to the lower chamber as a chemoattractant. The chambers were incubated at 37°C and 5% CO_2_ for 48 h. Then, fixed the cells on the lower surface in 100% methanol, then stained in 0.05% crystal violet. Five random fields were selected to count the number of cells for analysis. All experiments were performed in triplicate.

### 4.6 RNA sequencing

C4‐2 cells were treated with DMSO or 1 mM MLT for 48 h, and three duplicate samples were collected for RNA sequencing. The RNA sequencing was executed by OE Biotech. Using TRIzol reagent (Invitrogen), RNA was extracted. Using a TruSeq Stranded Total RNA Ribo‐Zero Gold kit, removed rRNA. Then reverse transcription was carried out to obtain cDNA, which was quality controlled by optical density and electrophoresis through agarose gel. The libraries were sequenced on an Illumina HiSeq X Ten platform. Generated 150 bp paired‐end reads. Using Trimmomatic, raw data of fastq format were firstly processed, and the low‐quality reads were removed to obtain the clean reads.^73^ About 80 M clean reads for each sample were retained for subsequent analyses. Mapped the clean reads to the human genome (GRCh38) using HISAT2.^74^ Differential expression analysis was carried out by using the DESeq R package (version: 1.18.0).^75^ DEGs were further analyzed based on a |LogFC| > 1 and a *p* value < 0.05.

### 4.7 LC‐MS/MS analysis

C4‐2 cells, treated with DMSO or 1 mM MLT for 48 h, were collected and stored at ‐80°C for the LC/MS analysis. The LC‐MS/MS detection and analysis were executed by Shanghai Lu Ming Biological Technology Co. Ltd. Identified the metabolites by using Progenesis QI (Waters Corporation, Milford) data processing software and public databases (http://www.hmdb.ca/ and http://www.lipidmaps.org/), and in‐house databases.

### 4.8 BODIPY staining

The cells or fresh frozen sections were fixed in 4% paraformaldehyde solution and incubated in the dark with BODIPY for 30 min and DAPI for 10 min. Five random fields were selected and analyzed using ImageJ software. All experiments were carried out implemented in triplicate.

### 4.9 Measurement of MLT, TGs, and T‐CHO

According to the MLT ELISA kit's instructions (Abcam, ab213978, USA), the levels of MLT in the plasma were measured. TG and T‐CHO contents in the cells and plasma were measured using a T‐CHO assay kit (Nanjing Jiancheng Bioengineering, cat. A111‐1‐1) and a TG assay kit (Nanjing Jiancheng Bioengineering, cat. A110‐1‐1), respectively, according to the manufacturers’ instructions. Blood collections (100 μl) were performed by caudal vein puncture, and blood was added to a procoagulant tube and centrifuged at 3000 rpm for 10 min at 4°C to obtain serum. The cells or tissues were lysed in PBS by sonication and centrifuged at 12,000 rpm for 10 min at 4°C to obtain extracts. The results were determined spectrophotometrically at 510 nm. A BCA kit was used to detect the protein concentration as a control.

### 4.10 Bisulfite genomic sequence PCR (BSP)

BSP was performed by Servicebio (Wuhan) according to the methods described previously (81). The UCSC Genome Browser (Human assembly hg38 from December, 2013) was used to predict the human CES1 promoter‐related CpG islands (CGIs); the primers for BSP were designed by using MethPrimer online software (http://www.urogene.org/methprimer/). Treated genomic DNA samples with sodium bisulfite, and PCR was performed. Bisulfite sequencing analysis was carried out using an ABI3730XL sequencing system.

### 4.11 Western blotting

Western blotting was performed according to the methods described previously (81). The primary antibodies included anti‐CES1 (1:2000, Proteintech, 16912‐1‐AP), anti‐MT1 (1:1000, Abcam, ab203038), anti‐SIRT1 (1:5000, Proteintech, 13161‐1‐AP), anti‐DNMT1 (1:1000, Abcam, ab188453), anti‐DNMT2 (1:1000, Proteintech, 19221‐1‐AP), anti‐DNMT3a (1:1000, Abcam, ab227823), anti‐DNMT3b (1:1000, Abcam, ab79822), anti‐DNMT3L (1:500, Proteintech, 14939‐1‐AP), anti‐PPARa (1:1000, Proteintech, 15540‐1‐AP), anti‐phospho‐PERK (Thr980) (1:1000, CST, 3179), anti‐ pospho‐eIF2α (Ser51) (1:1000, CST, 3398), anti‐ATF6 (1:1000, CST, 65880), anti‐Bip (1:1000, CST, 3177), anti‐IRE1a (1:1000, CST, 3294), anti‐p‐IRE1a (1:1000, Abcam, ab124945), anti‐Caspase 3 (1:1000, CST, 9662), anti‐CYP11A1 (1:1000, Proteintech, 13363‐1‐AP), anti‐STARD4 (1:1000, Abacam, ab202060), anti‐DHRSX (1:2000, Abcam, ab171736), anti‐HNF4A (1:2000, Abcam, ab92378), anti‐MGAT4C (1:1000, Proteintech, 17841‐1‐AP), anti‐SLC7A10 (1:1000, Novus, NBP2‐85763), anti‐IGFBP1 (1:1000, Proteintech, 13981‐1‐AP), anti‐ALDH1A2 (1:1000, Proteintech, 13951‐1‐AP), anti‐ACSBG2 (1:1000, Novus, NBP1‐54963), anti‐ZIC1 (1:3000, Abcam, ab134951), anti‐SLC38A4 (1:1000, Proteintech, 20857‐1‐AP), anti‐acetyl Lysine (1:300, abcam, ab190479), anti‐HDAC1 (1:1000, Abcam, ab109411), anti‐β‐actin (1:10000, Proteintech, 20536‐1‐AP), and anti‐GAPDH (1:10000, Proteintech, 10494‐1‐AP).

### 4.12 Immunohistochemical staining and TUNEL assays

The tissue array sections were processed as reported previously (81). The primary antibodies included anti‐CES1 (1:200, Proteintech, 16912‐1‐AP), anti‐Ki67 (1:20000, Proteintech, 27309‐1‐AP), anti‐PERK (1:50, Abcam, ab192591), anti‐caspase‐3 (1:200, Proteintech, 19677‐1‐AP), anti‐CYP11A1 (1:200, Proteintech, 13363‐1‐AP), and anti‐PSA (1:200, Proteintech, 10679‐1‐AP). The protein expression levels in the tissue sections were assessed based on the immunoreactive score (IRS). IRS scores of 0–1 indicated negative staining; scores of 2–3 indicated mild staining; scores of 4–8 indicated moderate staining, and scores of 9–12 indicated strong positive staining.

The TUNEL assay was performed using a TMR (red) TUNEL cell apoptosis detection kit (Servicebio, China) based on the manufacturer's instructions. Five random fields were selected to count the number of TUNEL‐positive cells, and the apoptosis index in each field was calculated based on the percentage of TUNEL‐positive cells relative to the total number of cells.

### 4.13 co‐IP assays

Using Triton lysis buffer supplemented with a freshly prepared protease inhibitor cocktail and PMSF, the cells were lysed for 30 min on ice. Centrifugated at 12,000 × g for 10 min at 4°C. Then 20 μl of protein A/G PLUS‐agarose (Santa Cruz, CA, USA) was added into 100 μl of the supernatant and incubated overnight with a primary anti‐DNMT1 antibody (1:250, Proteintech, China) at 4°C. Then, using cold Triton lysis buffer, the immunoprecipitate was washed three times and then boiled with 2 × SDS loading buffer for 10 min. Then, Western blot assay was carried out according to the protocol described above.

### 4.14 Quantitative real‐time PCR assays

Quantitative real‐time PCR was executed as previously described.^76^ The primer sequences for AR‐V7 were as follows: Forward, 5′‐CCATCTTGTCGTCTTCGGAAATGTTA‐3′, Reverse, 5′‐TTTGAATGAGGCAAGTCAGCCTTTCT‐3. The housekeeping gene was GAPDH: forward, 5′‐TCAAGAAGGTGGTGAAGCAG‐3′, reverse, 5′‐CGTCAAAGGTGGAGGAGTG‐3′. We evaluate relative expression based on ΔΔCT value and used the student's *t*‐test as statistical test.

### 4.15 ChIP assays

ChIP assays were carried out as described previously.^77^ The primer sequences for the CES1 promoter CGIs region were as follows: P1‐For: TGCCTTTATCCTGGCCACTC, P1‐Re: TGCCTTTATCCTGGCCACTC; P2‐For: TGAACATCCTGAGATCGCCC, P2‐Re: GATCCAGCCGTAAGGGGACT; P3‐For: CGGGGCACGACTTCCATATTA, P3‐Re: GGGCGATCTCAGGATGTTCA; P4‐For, CCGCACACACGAGAAATTTAGG, P4‐Re, CTCCGTGATCCAGCCGTAAG. Used anti‐DNMT1 antibody for IP, and normal IgG as a negative control.

### 4.16 Luciferase reporter assays

Luciferase reporter assays were executed as described previously.^78^ CES1 promoter (CES1‐WT) and blank (CES1‐MUT) vector plasmids and a DNMT1 overexpression vector and blank vector plasmid were purchased from GeneChem. According to the manufacturer's instruction, renilla luciferase was used for the normalization of the luciferase activity detected using the dual‐luciferase reporter assay kit (Promega, Madison, WI, USA).

### 4.17 Bioinformatics analysis

The mRNA expression of the genes in PCa tissues, adjacent normal tissues and the clinical data of the patients were obtained from The Cancer Genome Atlas (TCGA) database (http://www.cbioportal.org/public‐porta) and the GEO database (Gene Expression Omnibus, https://www.ncbi.nlm.nih.gov/geo/, namely, the Taylor GSE21034, Wallace Prostate GSE6956, and Yu Prostate GSE 6919 datasets). GSEA was performed to identify the pathways enriched with the gene set. We used C2: curated gene sets for GSEA. Based on *p* value (*p* < 0.05) and FDR (FDR < 0.25), we identify gene sets which are associated with CES1 in PCa TCGA dataset. Then based on CORE ENRICHMENT in the result of GSEA, we identify genes which are associated with CES1 in the gene set. These genes and the DEGs in RNA sequencing are intersected, then obtain the statistically significant genes which are associated with MLT/CES1 in PCa.

### 4.18 Systematic review and meta‐analysis

The literature search was executed independently by two investigators. We searched literature in the database Medline, PubMed, EMBASE, the Cochrane library, and CNKI, from inception to November 2020; selected appropriate studies for subsequent analyses, according to the inclusion criteria in Figures S1A and S1B. Using a standardized data extraction form, two investigators extracted information from each study independently. After quality assessment, statistics was performed in random model or fix effect model, using the software STATA 14.0.

### 4.19 Statistical analysis

Statistical analyses were performed using SPSS 22.0 (IBM, NY, USA) or GraphPad Prism 7.0 (GraphPad Software, San Diego, California, USA). A Kaplan–Meier analysis and log‐rank test were carried out to assess the survival of the patients. Statistical analyses were executed using Mann‐Whitney U test, Student's *t*‐test, paired Student's *t*‐test, and receiver operator characteristic curve. *p* values < 0.05 were considered statistically significant.

## CONFLICT OF INTEREST

The authors declare that they have no conflict of interest to this work.

## AUTHOR CONTRIBUTIONS

Ke Chen, Lijie Zhou, and Liang Wang conceived the idea. Lijie Zhou and Cai Zhang conceived and designed the experiments. Lijie Zhou, Hong Tao, and Haruhiko Sugimura performed the experiments. Lijie Zhou and Xiong Yang helped with animal experiments. Yaxin Hou and Zhaohui Chen helped to obtain clinical samples. Lijie Zhou, Lilong Liu, and Junyi Hu, analyzed the data. Lijie Zhou and Ke Chen wrote the manuscript. All authors reviewed and approved the manuscript.

## Supporting information

Supporting Information

Supporting Information
