# Supplementary material for: Melatonin inhibits lipid accumulation to repress prostate cancer progression by mediating the epigenetic modification of CES1
Source: Clin Transl Med. 2021 Jun 6;11(6):e449. doi: 10.1002/ctm2.449 (PMC8181204; doi:10.1002/ctm2.449)
Supplement: Supplementary file 2 — Supporting Information [file CTM2-11-e449-s001.pdf]

Melatonin inhibits lipid accumulation to repress prostate cancer progression by mediating the epigenetic modification of CES1

Lijie Zhou[1,2 #], Cai Zhang[3], Xiong Yang[1], Lilong Liu[1,2], Junyi Hu[1,2], Yaxin Hou[1,2], Hong Tao[4], Haruhiko Sugimura[4], Zhaohui Chen[1], Liang Wang[1 *], Ke Chen[1,2 *]

[1]Department of Urology, Union Hospital, Tongji Medical College, Huazhong University of Science and Technology, Wuhan 430022, China;

[2]Shenzhen Huazhong University of Science and Technology Research Institute, Shenzhen, China;

[3]Department of Clinical Laboratory, the First Affiliated Hospital of Zhengzhou University, Zhengzhou, Henan, China;

[4]Department of Tumor Pathology, Hamamatsu University School of Medicine, Hamamatsu, Shizuoka, Japan.

Running Head: Melatonin repress prostate cancer progression

Figures: 9 main figures; 2 supplementary tables; 6 supplementary figures

*Corresponding author:

Liang Wang, Department of Urology, Union Hospital, Tongji Medical College, Huazhong University of Science and Technology, 1277 Jiefang Avenue, Wuhan, 430022, Hubei Province, China; wanglianguh@163.com;

Ke Chen, Department of Urology, Union Hospital, Tongji Medical College, Huazhong University of Science and Technology, 1277 Jiefang Avenue, Wuhan, 430022, Hubei Province, China; E-mail: shenke@hust.edu.cn.

Supplementary Figure Legends

Figure S1. MLT inhibits lipid accumulation in the blood and in PCa cells. (A-C) Flow diagram of the studies assessed by the systematic review and meta-analysis. (D) AR-V7 mRNA expression in prostate cancer cell lines as determined by real-time PCR. (E-F) IHC assays and statistical analysis of the IRS scores were performed to detect the levels of Ki67 in the Rm-1 cell xenograft tumor tissues and and TUNEL assays were performed to detect the apoptosis rate of the tumor tissue cells. Representative images are shown. (G) IHC staining of Rabbit IgG (R-IgG) as negative control in the paraffin-embedded prostate tissue arrays. T: prostate tumor tissue; N: normal prostate tissue.

Figure S2. CES1 is significantly regulated in melatonin-treated PCa cells (A) Volcano map of up- and downregulated genes in C4-2 cells treated with MLT (1 mM). And the number of DEGs in the C4-2 cells treated with MLT (1 mM). (B) C4-2 and 22RV1 cells were transfected with siMT1. MT1 protein levels were determined by Western blot analysis. Densitometry and statistical analysis. Representative images are shown. (C) Heat map of differential lipid metabolites in the C4-2 cells treated with MLT based on those in the control cells (DMSO treated) according to the results of the LC-MS/MS lipidomic assay. Only statistically significant changes ($p < 0.05$, VIP > 1) are presented. (D) C4-2 and 22RV1 cells were treated with 1 mM MLT for 48 h. CES1, DHRSX, HNF4A, SLC7A10, IGFBP1, ALDH1A2, ACSBG2, ZIC1, and SLC38A4 protein levels were determined by Western blotting. Densitometry and statistical analysis. (E) After transfection with siMT1, C4-2 and 22RV1 cells were treated with MLT (1 mM) for 48 h. Western blot analysis was performed to determine the levels of CES1 protein expression. Densitometry and statistical analysis. Representative images are shown.

Figure S3. CES1 is a key gene in PCa, and associated with lipid accumulation. MLT inhibited cell invasion and reversed enzalutamide resistance by CES1. (A) The receiver operating characteristic (ROC) curves of CES1 in PCa. (B) Multivariate analyses of clinical pathological factors of the disease. (C) Kaplan–Meier analysis of the associations of the level of CES1 mRNAs with OS in the TCGA PCa (n = 497) and

Taylor databases (n = 150). (D) GSEA of the correlation of the CES1 mRNA levels and lipid accumulation-related pathways in a TCGA PCa cohort. (E) After transfection with siMT1, C4-2 and 22RV1 cells were treated with MLT (1 mM) for 48 h. Western blot analysis was performed to determine the levels of CES1 protein expression. Densitometry and statistical analysis. Representative images are shown. (F) Cell migration was evaluated in C4-2 and 22RV1 cells with CES1-knockout by Transwell assays. The results are presented as the mean ± SEM of 3 independent experiments. (G) GSEA of the correlation of the CES1 mRNA levels and lipid metabolism-related pathways in the TCGA PCa cohort. (H) TG and T-CHO contents were measured in C4-2 and C4-2-ENZR cells. The graphs represent the means ± SEM. (I) The mRNA levels of PPARa-related genes (CPT1C and ME1) are dramatically upregulated in the C4-2 cells treated with MLT. The mRNA levels of steroid hormone metabolism-related genes (CYPA11A1 and STARD4) were downregulated in C4-2 cells treated with MLT. (J) GSEA of the correlations of the CES1 mRNA levels and cholesterol metabolism-related pathways in a TCGA PCa cohort. (K) WB assays were used to measure the CES1 expression in the C4-2-ENZR cells treated with MLT.

Figure S4. MLT regulates the methylation of the CES1 promoter via SIRT1-mediated DNMT1 deacetylation. (A) The sequences of CGIs in the CES1 promoter regions were examined experimentally by BSP. (B) The sequence of DNMT1 binding to DNA is predicted by CisBP. (C) C4-2 and 22RV1 cells were transfected with siSIRT1, and the control siRNA (siNC). SIRT1 protein levels were determined by WB. Densitometry and statistical analysis. Representative images are shown. (D) Densitometry and statistical analysis of the data of Figure 7H.

Figure S5. MLT inhibition of tumor growth and reversal of ENZ resistance of CPRC are mediated by CES1 expression in vivo. (A) IHC and TUNEL assays were performed to detect the levels of CES1, Ki67, PPARa, PERK, and caspase-3 in the C4-2 cell xenograft tumors and the apoptosis rate of the tumor cells. Representative images are shown. IHC staining of Rabbit IgG (R-IgG) as negative control. (B) IHC and TUNEL assays were performed to detect the levels of CES1, Ki67, CYP11A1, and PSA in the C4-2-ENZR cell xenograft tumor tissues and the apoptosis rate of the tumor tissue

cells. IHC staining of Rabbit IgG (R-IgG) as negative control. Representative images are shown. (C) 22RV1 cells were transfected with a siRNA targeting PPARα, CYP11A1, PERK, Caspase 3 or PSA, with a control siRNA (siNC). Western blot was performed to test the antibody validation.

Figure S6. MLT inhibited tumor growth and reversed of ENZ resistance in animal CRPC models with a circadian rhythm disorder. (A-B) IHC assays and statistical analysis of the IRS scores of IHC staining were performed to detect the levels of Ces1, Ppara, Perk, caspase-3, Cyp11a1, and Psa in the Rm-1 cell xenograft tumor tissues. IHC staining of Rabbit IgG (R-IgG) as negative control.Representative images are shown. Statistical analysis of the IRS scores. (C) IHC staining of Rabbit IgG (R-IgG) as negative control.

Table S1

Review1: Influence of MLT levels on the risk of prostate cancer

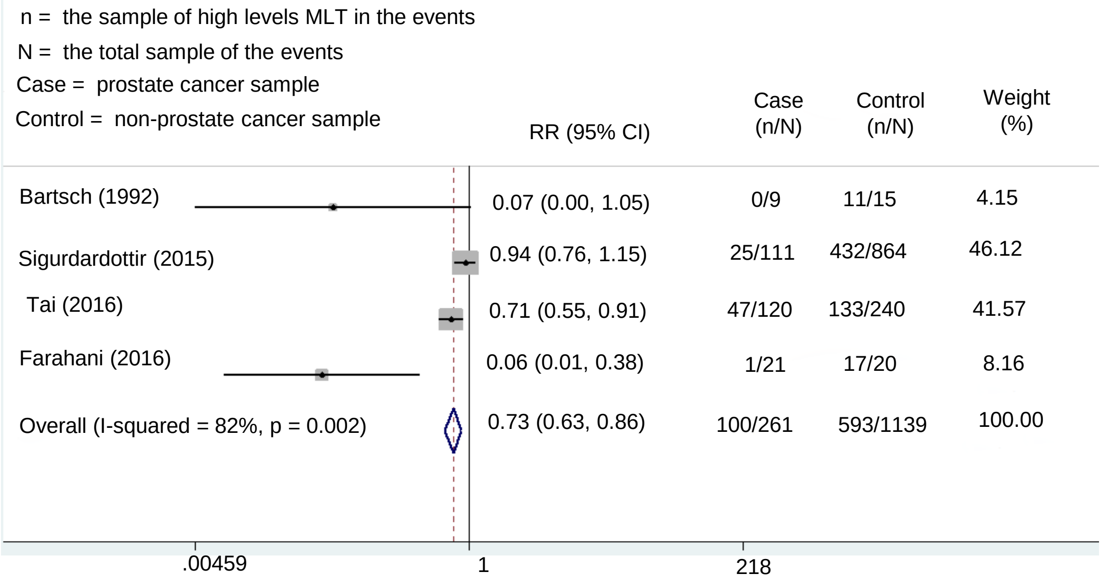

Table S2

Review 1: the levels of MLT and T-CHO

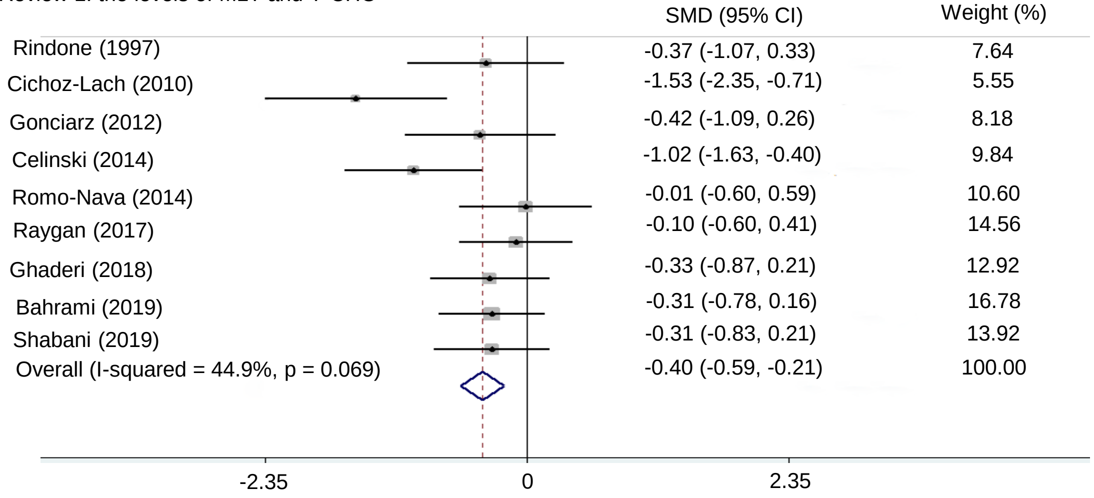

Review 2: the levels of MLT and LDL

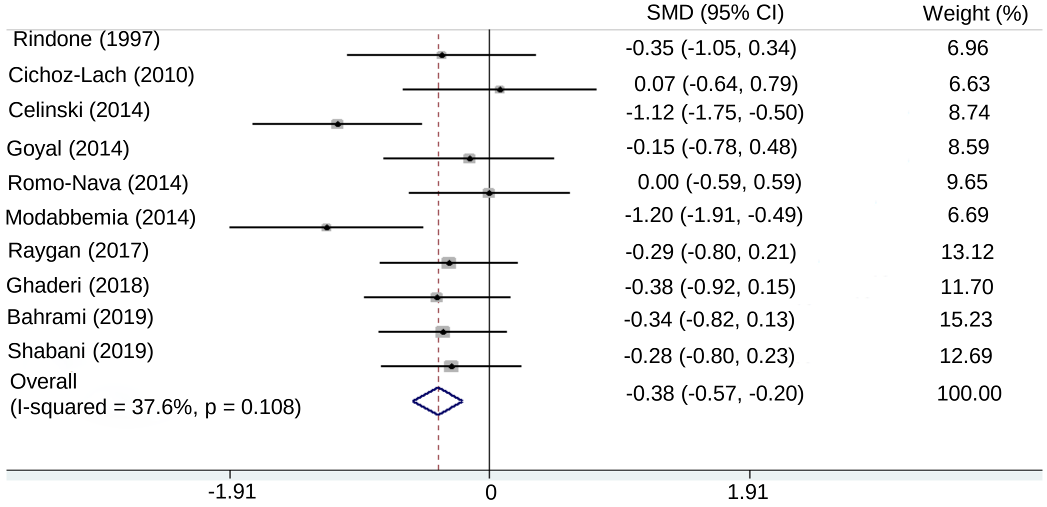

Review 3: the levels of MLT and HDL

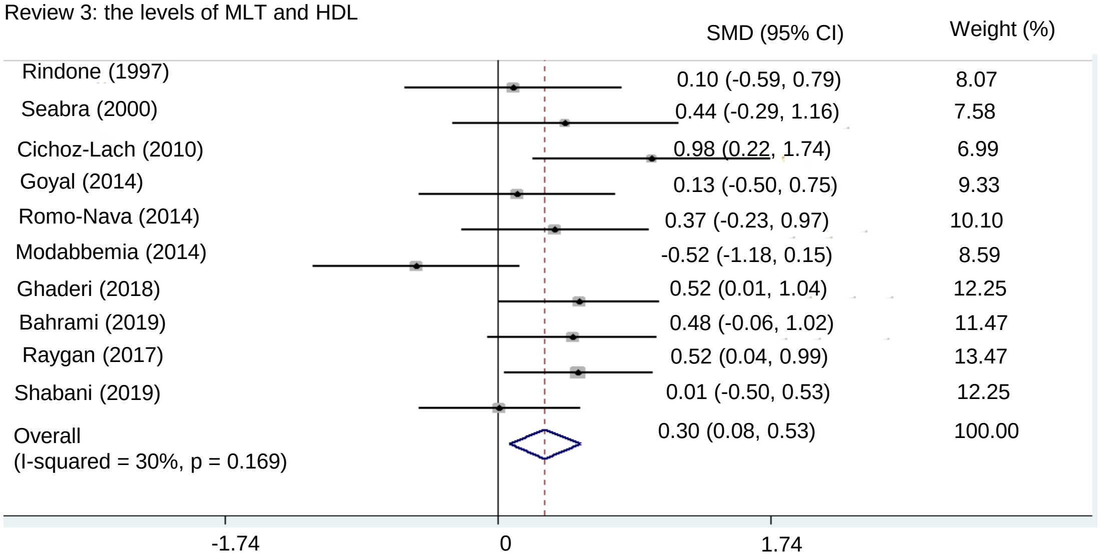

Figure S1

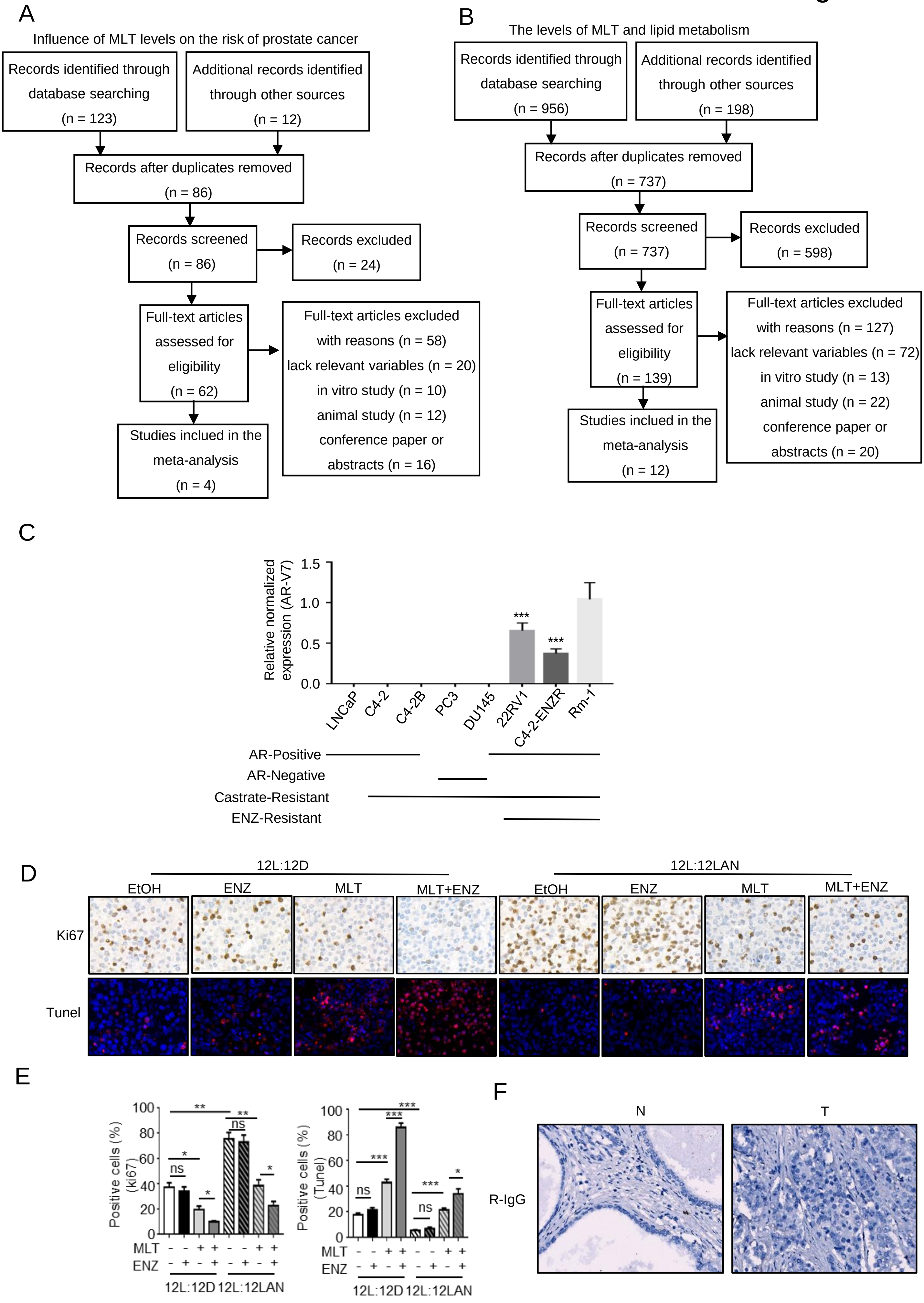
A
Influence of MLT levels on the risk of prostate cancer
Records identified through database searching (n = 123)
Additional records identified through other sources (n = 12)
Records after duplicates removed (n = 86)
Records screened (n = 86)
Records excluded (n = 24)
Full-text articles assessed for eligibility (n = 62)
Full-text articles excluded with reasons (n = 58)
lack relevant variables (n = 20)
in vitro study (n = 10)
animal study (n = 12)
conference paper or abstracts (n = 16)
Studies inclued in the meta-analysis (n = 4)
B
The levels of MLT and lipid metabolism
Records identified through database searching (n = 956)
Additional records identified through other sources (n = 198)
Records after duplicates removed (n = 737)
Records screened (n = 737)
Records excluded (n = 598)
Full-text articles assessed for eligibility (n = 139)
Full-text articles excluded with reasons (n = 127)
lack relevant variables (n = 72)
in vitro study (n = 13)
animal study (n = 22)
conference paper or abstracts (n = 20)
Studies inclued in the meta-analysis (n = 12)
C
Relative normalized expression (AR-V7)
1.5
1.0
0.5
0.0
***
***
LNCaP
C4-2
C4-2B
PC3
DU145
22RV1
C4-2-ENZR
Rm-1
AR-Positive
AR-Negative
Castrate-Resistant
ENZ-Resistant
D
12L:12D
12L:12LAN
EtOH
ENZ
MLT
MLT+ENZ
Ki67
Tunel
E
Positive cells (%) (ki67)
Positive cells (%) (Tunel)
100
80
60
40
20
0
**
**
ns
*
*
*
***
***
***
***
*
ns
MLT - - + + - - + +
ENZ - + - + - + - +
12L:12D 12L:12LAN
F
N
T
R-IgG

Figure S2

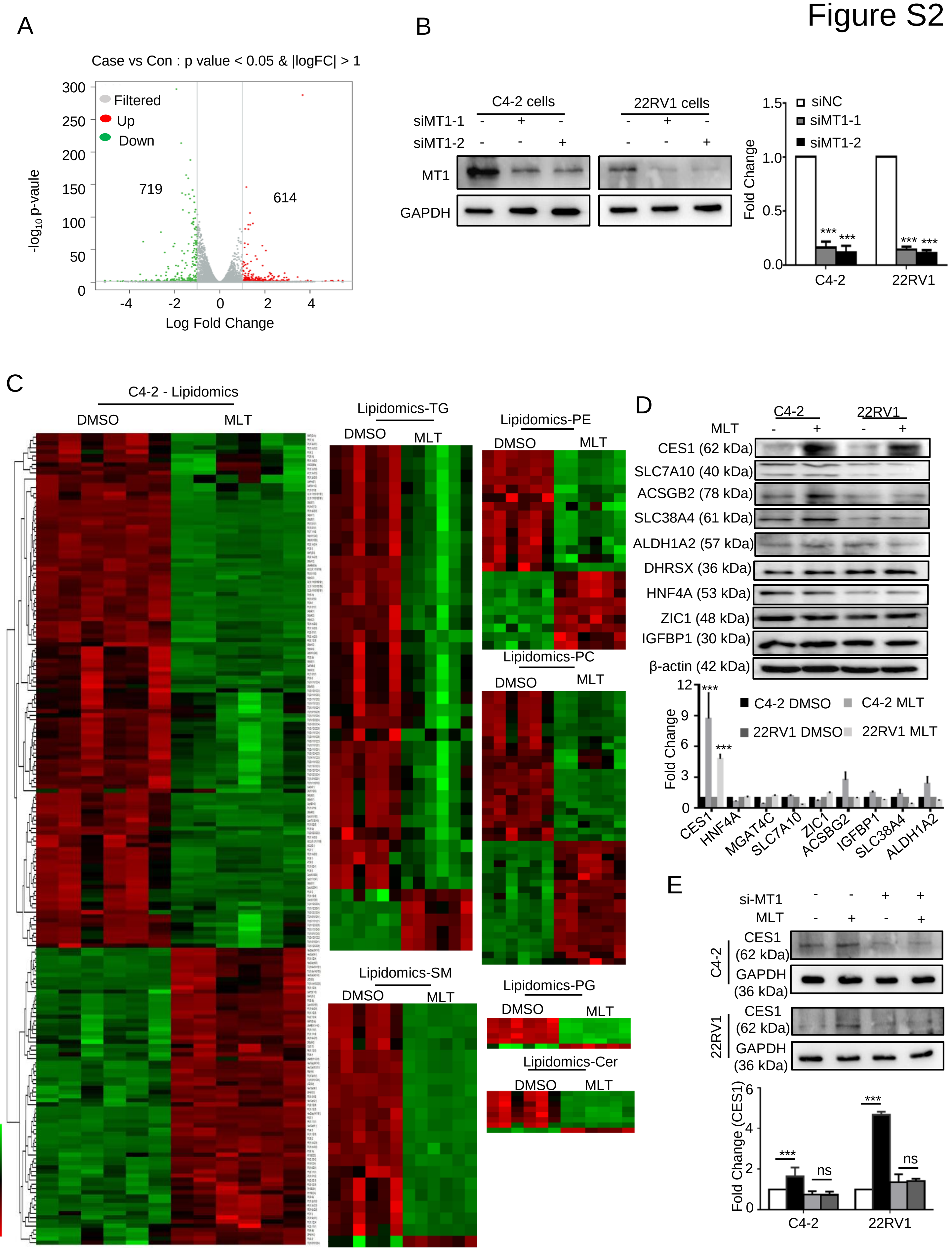

TG (Triglyceride)
PE (Phosphatidylethanolamine)
PC (Phosphatidylcholine)
SM (Sphingomyelin)
Cer (Ceramide)

Figure S3

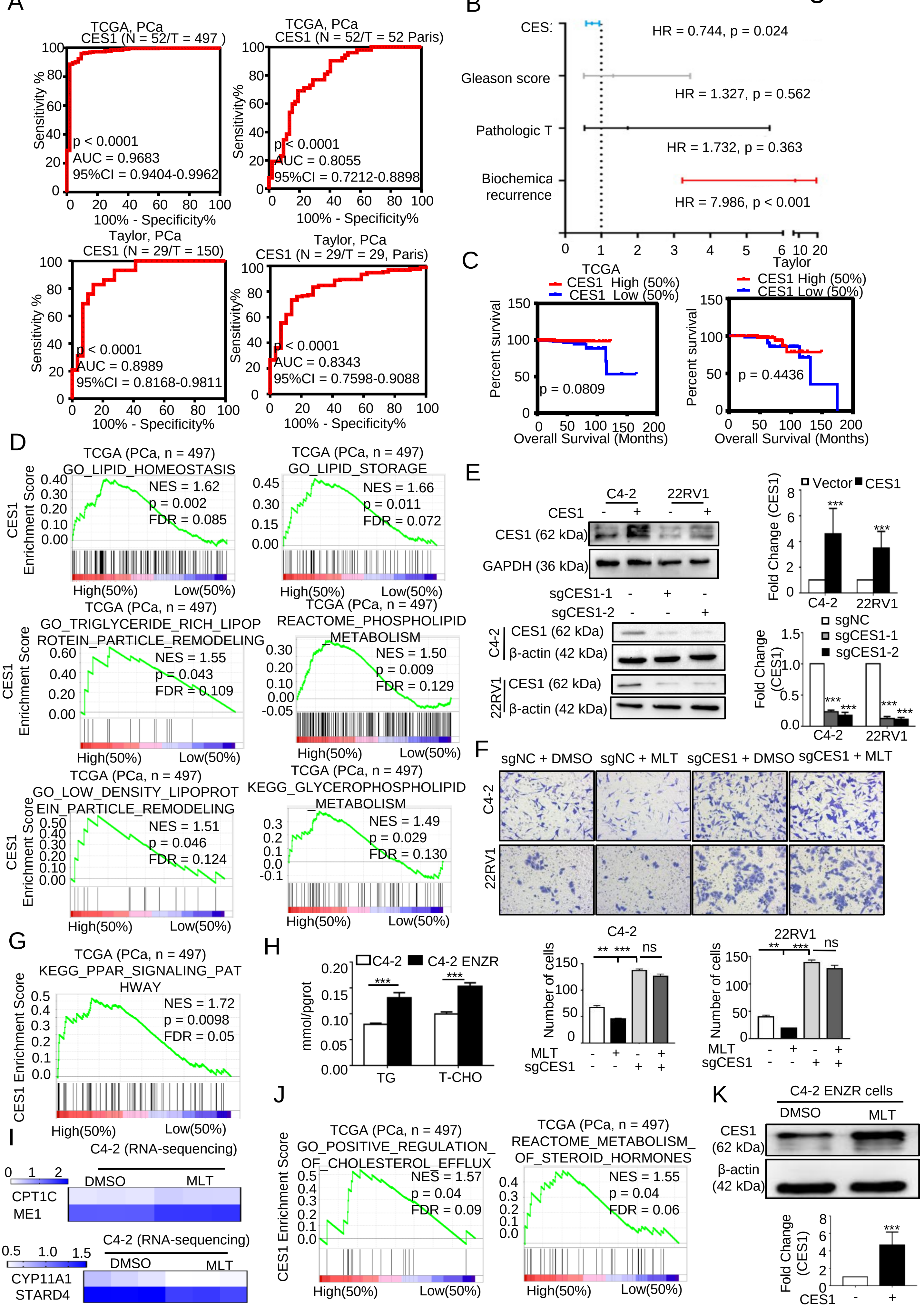

A
TCGA, PCa
CES1 (N = 52/T = 497)
p < 0.0001
AUC = 0.9683
95%CI = 0.9404-0.9962
CES1 (N = 52/T = 52 Paris)
AUC = 0.8055
95%CI = 0.7212-0.8898
Taylor, PCa
CES1 (N = 29/T = 150)
AUC = 0.8989
95%CI = 0.8168-0.9811
CES1 (N = 29/T = 29, Paris)
AUC = 0.8343
95%CI = 0.7598-0.9088
Sensitivity %
100% - Specificity%
B
Gleason score
Pathologic T
Biochemical recurrence
HR = 0.744, p = 0.024
HR = 1.327, p = 0.562
HR = 1.732, p = 0.363
HR = 7.986, p < 0.001
Taylor
C
TCGA
CES1 High (50%)
CES1 Low (50%)
Percent survival
p = 0.0809
Overall Survival (Months)
p = 0.4436
D
TCGA (PCa, n = 497)
GO_LIPID_HOMEOSTASIS
NES = 1.62
p = 0.002
FDR = 0.085
GO_LIPID_STORAGE
NES = 1.66
p = 0.011
FDR = 0.072
GO_TRIGLYCERIDE_RICH_LIPOPROTEIN_PARTICLE_REMODELING
NES = 1.55
p = 0.043
FDR = 0.109
REACTOME_PHOSPHOLIPID_METABOLISM
NES = 1.50
p = 0.009
FDR = 0.129
GO_LOW_DENSITY_LIPOPROTEIN_PARTICLE_REMODELING
NES = 1.51
p = 0.046
FDR = 0.124
KEGG_GLYCEROPHOSPHOLIPID_METABOLISM
NES = 1.49
p = 0.029
FDR = 0.130
CES1 Enrichment Score
High(50%)
Low(50%)
E
C4-2
22RV1
CES1
CES1 (62 kDa)
GAPDH (36 kDa)
Vector
Fold Change (CES1)
sgCES1-1
sgCES1-2
β-actin (42 kDa)
sgNC
F
sgNC + DMSO
sgNC + MLT
sgCES1 + DMSO
sgCES1 + MLT
G
KEGG_PPAR_SIGNALING_PATHWAY
NES = 1.72
p = 0.0098
FDR = 0.05
H
C4-2 ENZR
mmol/pgrot
TG
T-CHO
Number of cells
MLT
ns
I
C4-2 (RNA-sequencing)
DMSO
CPT1C
ME1
CYP11A1
STARD4
J
GO_POSITIVE_REGULATION_OF_CHOLESTEROL_EFFLUX
NES = 1.57
p = 0.04
FDR = 0.09
REACTOME_METABOLISM_OF_STEROID_HORMONES
NES = 1.55
p = 0.04
FDR = 0.06
K
C4-2 ENZR cells
CES1 (62 kDa)
β-actin (42 kDa)

Figure S4

A

GATTTGGATTTGGAGTAGTTGAGTTCGGTTAGTTTGGTTCGCGCGGAGACGTAGGGAGGGTGAATATTT
TGAGATCGTTTTGTATTGTTCGTTTAAATGTAGGTCGATAGTCGGTTTATTTTAGGTCGTATATACGAGAA
ATTTAGGTAGTTTAGTTTGTTGTTTTTTAATTAGTTTTTTTACGGTTGGATTACGGAGAGCGGTTTAATATA
ATATTAAGAGTATCGATTTTGGAGTTAGAGTGCGTGGGTTCGAGTTTTCGTTGGGCGTTTTTTCGTTGTG
TGTTTTTAGGTAAGGGATTGCGTTATTTCGTGTTTGGGTTATTTTTTTTTCGGGTGGTAGTGTTGTTTATT

B

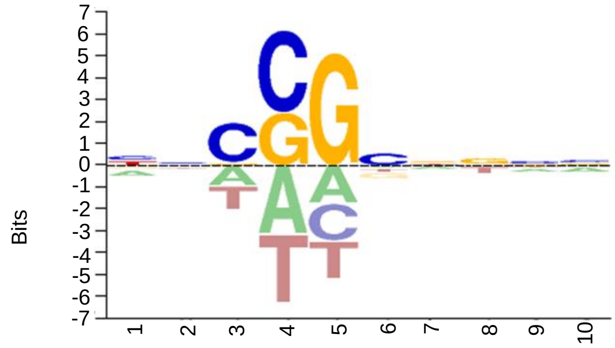

C

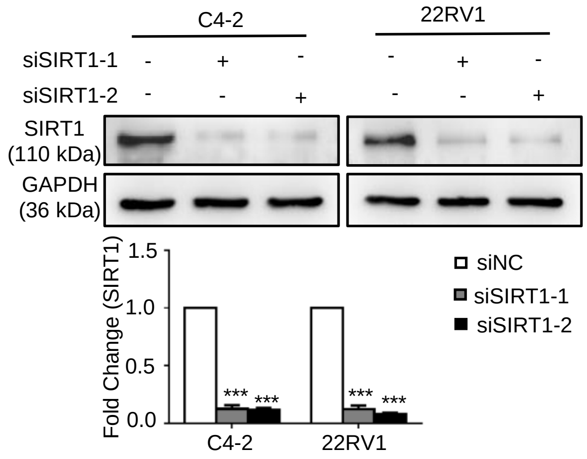

D

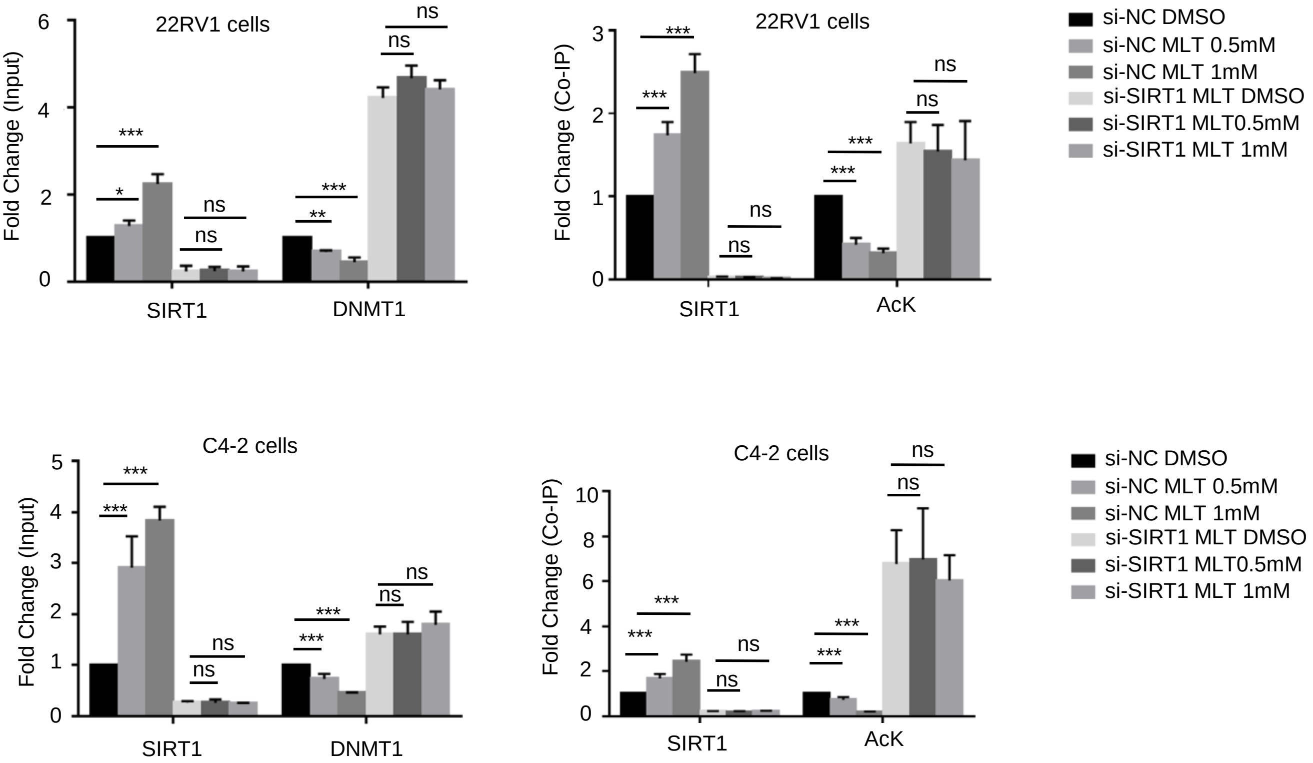

Figure S5

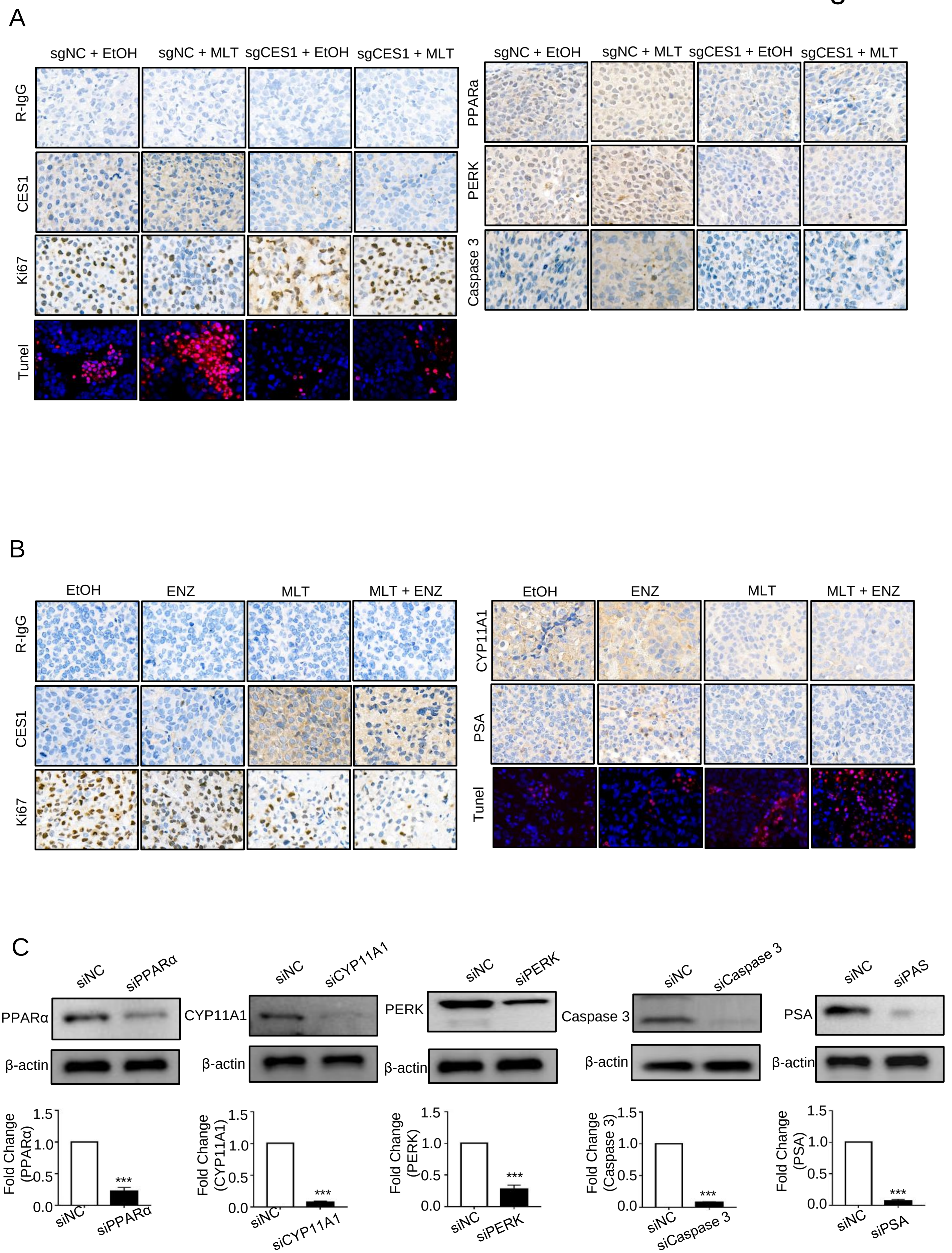
A
sgNC + EtOH
sgNC + MLT
sgCES1 + EtOH
sgCES1 + MLT
R-IgG
CES1
Ki67
Tunel
PPARa
PERK
Caspase 3
B
EtOH
ENZ
MLT
MLT + ENZ
CYP11A1
PSA
C
siNC
siPPARα
PPARα
β-actin
siCYP11A1
CYP11A1
siPERK
PERK
siCaspase 3
Caspase 3
siPAS
PSA
Fold Change (PPARα)
Fold Change (CYP11A1)
Fold Change (PERK)
Fold Change (Caspase 3)
Fold Change (PSA)
1.5
1.0
0.5
0.0
***
siPSA

Figure S6

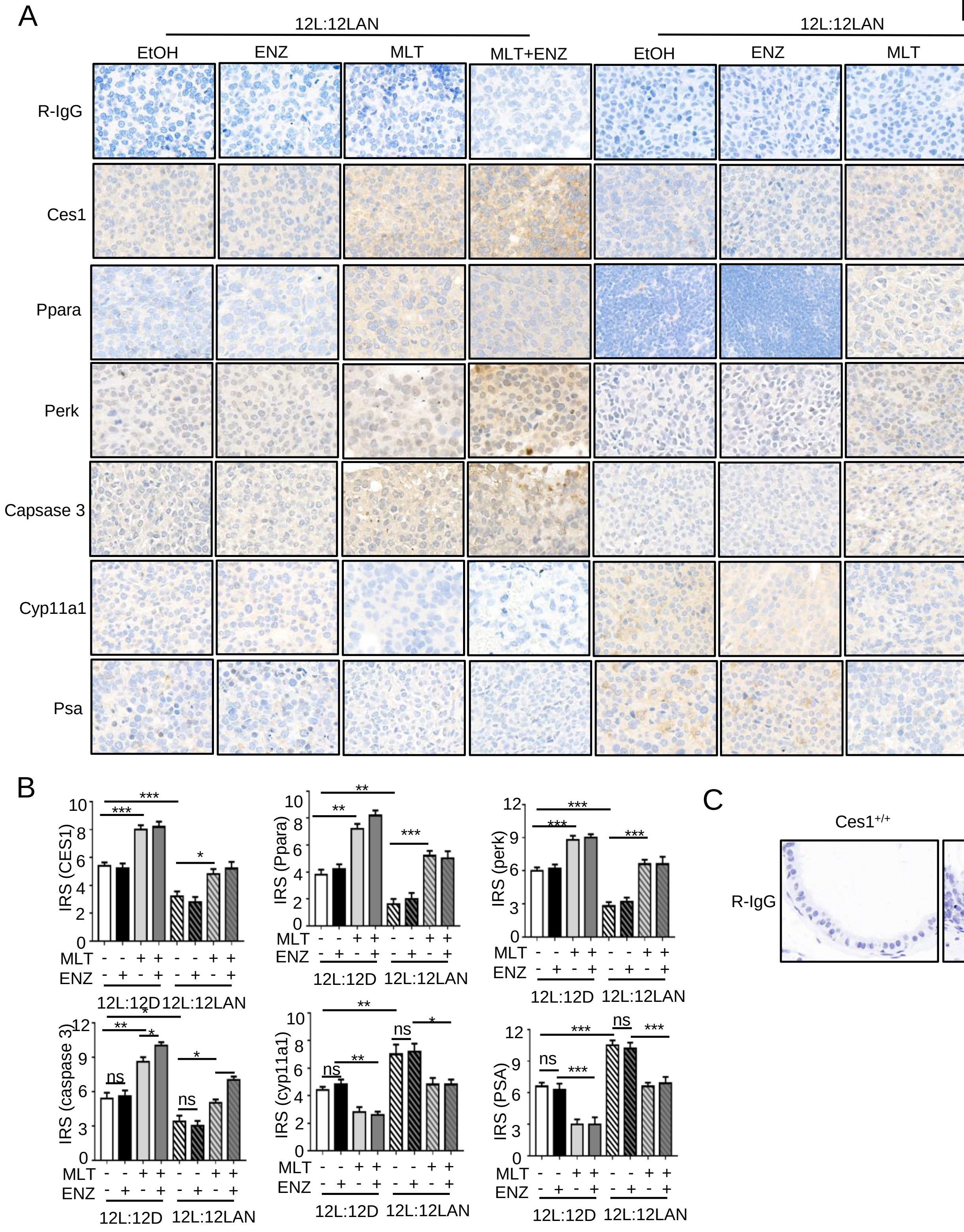

A
12L:12LAN
12L:12LAN
EtOH
ENZ
MLT
MLT+ENZ
EtOH
ENZ
MLT
MLT+ENZ
R-IgG
Ces1
Ppara
Perk
Capsase 3
Cyp11a1
Psa
B
IRS (CES1)
IRS (Ppara)
IRS (perk)
IRS (caspase 3)
IRS (cyp11a1)
IRS (PSA)
MLT
ENZ
12L:12D
12L:12LAN
ns
C
Ces1+/+
Ces1-/-
R-IgG
